# Incorporating thermal co-evaporation in current-matched all-perovskite triple-junction solar cells†

**DOI:** 10.1039/d4el00012a

**Published:** 2025-01-21

**Authors:** Terry Chien-Jen Yang, Taeheon Kang, Melissa Fitzsimmons, Guadalupe Vega, Yang Lu, Leo Rosado, Alberto Jiménez-Solano, Linfeng Pan, Szymon J. Zelewski, Jordi Ferrer Orri, Yu-Hsien Chiang, Dengyang Guo, Zher Ying Ooi, Yutong Han, Weidong Xu, Bart Roose, Caterina Ducati, Sol Carretero Palacios, Miguel Anaya, Samuel D. Stranks

**Affiliations:** a Department of Chemical Engineering and Biotechnology, University of Cambridge Cambridge CB3 0AS UK; b Department of Physics, Cavendish Laboratory, University of Cambridge Cambridge CB3 0HE UK sds65@cam.ac.uk; c Departamento Física de la Materia Condensada, Instituto de Ciencia de Materiales de Sevilla, Universidad de Sevilla−CSIC Calle Américo Vespucio 49 41092 Sevilla Spain; d Instituto de Ciencia de Materiales de Madrid, ICMM-CSIC 28049 Madrid Spain; e Departamento de Física, Universidad de Córdoba Edificio Einstein (C2), Campus de Rabanales 14071 Córdoba Spain; f Department of Experimental Physics, Faculty of Fundamental Problems of Technology, Wrocław University of Science and Technology 50-370 Wrocław Poland; g Department of Materials Science and Metallurgy, University of Cambridge Cambridge CB3 0FS UK

## Abstract

Thermal co-evaporation of halide perovskites is a solution-free, conformal, scalable, and controllable deposition technique with great potential for commercial applications, particularly in multi-junction solar cells. Monolithic triple-junction perovskite solar cells have garnered significant attention because they can achieve very high efficiencies. Nevertheless, challenges arise in fabricating these devices, as they require multiple layers and precise current matching across complex absorber stacks. Here we demonstrate a current-matched monolithic all-perovskite p–i–n triple-junction solar cell enabled by controlled thermal co-evaporation of various absorber layers in the stack. The top and middle subcells were fabricated by developing optimized thermally co-evaporated Cs_0.3_FA_0.7_Pb(I_0.56_Br_0.44_)_3_ (1.80 eV bandgap) and FAPbI_3_ (1.53 eV) perovskites, respectively, while the bottom subcell employed a solution-processed Cs_0.25_FA_0.75_Pb_0.5_Sn_0.5_I_3_ (1.25 eV) perovskite. By optimising absorber thicknesses and compositions through optical modelling, we achieve excellent current matching between the top (9.6 mA cm^−2^), middle (9.3 mA cm^−2^), and bottom subcells (9.0 mA cm^−2^), achieving an overall efficiency of 15.8%. Optical modelling simulations suggest that current matching and efficiency up to 11.4 mA cm^−2^ and 37.6% respectively could be attainable using the latest interlayer materials. This work highlights the potential of scalable vapour-based deposition techniques for advancing multi-junction perovskite-based solar cells, paving the way for future developments in this field.

## Abstract 2

Thermal co-evaporation of halide perovskites is a solution-free, conformal, scalable, and controllable deposition technique with great potential for commercial applications, particularly in multi-junction solar cells.

## Graphical abstract

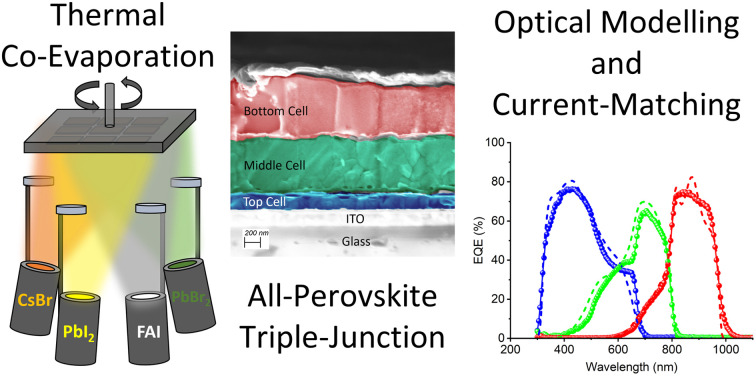

**Broader context**

Single-junction crystalline silicon solar cells, the dominant technology in photovoltaics today, are nearing their maximum theoretical efficiency of 29.4%. To continue to reduce the cost of solar energy, either in terms of $ per m^2^ or $ per W, higher efficiency is essential. One of the most promising and well-established strategies to exceed the single-junction efficiency limit is by stacking absorbers with varying bandgaps in a multi-junction cell. Bandgap tunable perovskite materials have the potential to be the next mainstream photovoltaic technology. In particular, multi-junction perovskite-based solar cells have garnered significant attention because they can achieve very high efficiencies. One method of depositing perovskite films is thermal co-evaporation which is a vacuum technique that is solution-free, conformal, scalable, and highly thickness controllable. Thus, it is very attractive for commercial applications especially in monolithic multi-junction perovskite-based solar cells where current matching between the subcells is critical. We demonstrate a highly current-matched monolithic all-perovskite triple-junction solar cell enabled by controlled thermal co-evaporation of various perovskite absorber layers in the stack and aided by feedback through optical modelling simulations using the refractive index data from these materials. This research paves the way for expanding the use of vacuum-deposition thermal co-evaporation techniques in perovskite-based multi-junction research and development.

## Introduction

Perovskite solar cells (PSCs) have become one of the most popular research topics in the field of photovoltaics due to their high-efficiency, bandgap tunability, low cost, and potential for commercialisation. The power conversion efficiency (PCE) of single-junction PSCs has increased from 3.8% (2009) to 26.7% (2024),^1^ which can be compared to the theoretical Shockley–Queisser (SQ) single-junction efficiency limit of 33%.^2,3^ Multi-junction (MJ) devices using various bandgap materials can achieve higher efficiency through better utilization of the solar spectrum by reducing both the above bandgap thermalization and below bandgap non-absorbed losses. Intensive efforts with double-junction monolithic tandems for both perovskite–perovskite^4–15^ and perovskite–silicon^16–28^ tandem solar cells have been widely demonstrated, with reported efficiency up to 34.6%.^1^ However, only a handful of 2-terminal monolithic perovskite-based triple-junction devices, either perovskite–perovskite–perovskite^29–32^ (PPP), perovskite–perovskite–silicon,^33–40^ and even perovskite–perovskite–organic,^41^ have been reported thus far. Hörantner *et al.*^42^ in 2017 reported through modelling studies that perovskite-based triple-junctions could potentially reach practical efficiencies up to 39%. Drawing reference to another existing photovoltaic (PV) technology, the record for the triple-junction III–V solar cell at 1-Sun AM1.5 has already reached 39.5%.^43,44^ The record device consists of carefully engineered III–V materials with an ideal bandgap combination of 1.88, 1.33, and 0.92 eV for the top, middle, and bottom subcells respectively, albeit with expensive absorber layers. Nevertheless, experimentally reported efficiencies of 2-terminal monolithic PPP and perovskite–perovskite–silicon triple-junction solar cells are only at 24.3% (ref. 32) and 27.6% (ref. 40) (31.5% (ref. 45) for 4-terminal), respectively, and far from their double-junction counterparts. The first reason is the complexity in terms of the number of functional layers that need to be deposited. A triple-junction solar cell requires a minimum of about 14 functional layers, which means it takes significant time and resources to even fabricate a single batch of devices, leading to slower feedback cycles than lower junction number analogues. Furthermore, if there is an issue with any of the layers, for example the uniformity (both vertically and laterally), composition, or thickness, then the final performance of the triple-junction device would be suboptimal. Initial efforts have been made to allow identification of problematic layers in MJ devices,^46^ but more work is needed in this area. The second reason is the stringent current matching criteria necessary for monolithic (series-connected) MJ solar cells. In a monolithic perovskite–perovskite tandem device, bandgap and current matching is required between only two absorbers. In a triple-junction PPP device, three absorbers must be optimally matched in terms of bandgap and current, creating more degrees of freedom (see ESI Note 1†). This makes precise current-matching more challenging, requiring judicious optimisation and a deep understanding of the device stack, such as through advanced optical and electrical modelling. A third challenge involves material compatibility, particularly during the deposition and annealing of subsequent functional layers, where process conditions must align without degrading the performance of underlying layers. These challenges are worth tackling and solving them will ultimately allow all-perovskite triple-junction device efficiencies to surpass their tandem counterparts (see ESI Note 2 and Table 1† for existing devices in literature).

Thermal co-evaporation is a conformal, upscalable, and highly thickness-controllable vacuum deposition technique for fabricating perovskite films.^47–50^ Such vapour-based techniques are very promising for the industrial commercialisation of MJ perovskite solar cell technology as seen with other thin film technologies including (single-junction) CdTe. The multi-source co-evaporation method^13,51–54^ has the advantage of fine tuning of the material composition and thus bandgap. This technique was pioneered for halide perovskites by the likes of Snaith *et al.*^55^ and Bolink *et al.*,^47,56^ but to date far less effort has been made on thermal evaporation than solution processed equivalents. Similarly, the majority of perovskites used in MJ solar cells to date have been solution-processed, with only a few notable exceptions demonstrating co-evaporated perovskite layers in tandem solar cells.^6,13,52,57,58^ The current state-of-the-art 24.3% monolithic triple-junction PPP solar cell made by Wang *et al.*^32^ was fabricated using solution-processed perovskites for all three of the subcells. For tandem devices where evaporated perovskites have been used, the record for perovskite–perovskite and perovskite–silicon are 24.1% (ref. 13) and 24.6% (ref. 59), respectively. So far, there has been no demonstration of a triple-junction perovskite solar cell made with thermal co-evaporation.

In this work, we first demonstrate an optimized thermally co-evaporated FAPbI_3_ perovskite composition, which has a suitable bandgap of 1.53 eV as the middle subcell in our triple-junction device. We then present the fabrication of a monolithic triple-junction PPP solar cell using thermally co-evaporated perovskite top and middle layers. This technique enables precise control over bandgap and thicknesses, driven by optical modelling and experimental feedback. By optimising the deposition process, we achieved exceptional current-matching across the top, middle, and bottom subcells, culminating in a short-circuit current (*J*_SC_) of 9.3 mA cm^−2^, among the highest reported for triple-junction PPP solar cells.

## Results and discussion

### Optimisation of a co-evaporated FAPbI_3_ middle cell

**Fig. 1:**
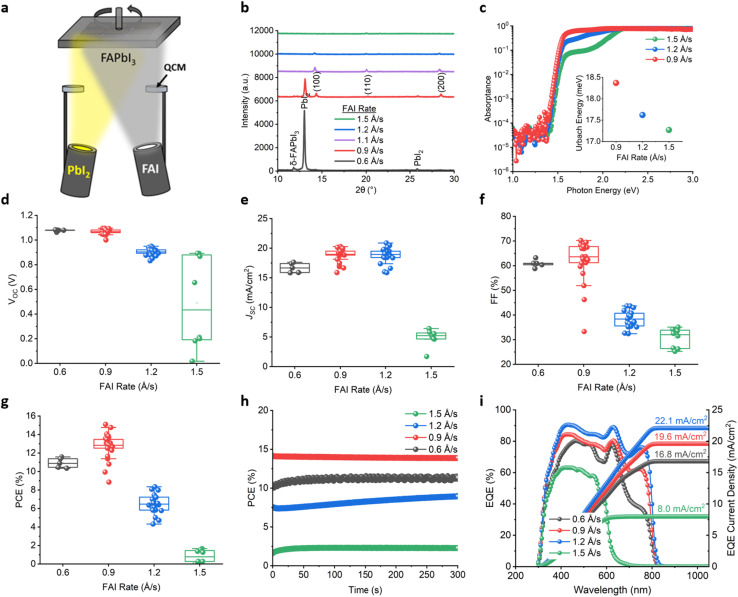
Thermally co-evaporated FAPbI_3_ absorber and solar cell characterisation. (a) Schematic of the FAPbI_3_ thermal co-evaporation process. (b) XRD patterns of various FAPbI_3_ films deposited on glass/ITO/MeO-2PACz. Note, the ratio between the FAI : PbI_2_ is represented by the evaporation rate of the FAI source, where the PbI_2_ source is fixed at 0.6 Å s^−1^, (c) PDS of FAPbI_3_ with different ratios deposited on fused silica substrates (inset: Urbach energy values), (d and g) PSC photovoltaic data (based on 2 batches of devices with a minimum of 3 devices (8 cells each) per rate) of the devices with the structure glass/ITO/MeO-2PAC/FAPbI_3_/C60/BCP/Cu, where (d) *V*_OC_, (e) *J*_SC_, (f) FF, (g) PCE, (h) *J*–*V* stability of four representative devices, (i) EQE with integrated EQE-*J*_SC_ of four representative devices.

In our previous work on thermal co-evaporation,^13^ we demonstrated wide-bandgap (up to 1.80 eV) Cs_0.3_FA_0.7_Pb(I_*x*_Br_1−*x*_)_3_ perovskites which were used as a top cell for a 2-terminal perovskite–perovskite tandem solar cell. Here we selected a nominal perovskite composition of Cs_0.3_FA_0.7_Pb(I_0.56_Br_0.44_)_3_ (1.80 eV) as a reliable base process for the top wide-bandgap absorber for our final PPP triple-junction solar cell. Thermal co-evaporation of the Cs_0.3_FA_0.7_Pb(I_*x*_Br_1−*x*_)_3_ perovskite required a 10% PbI_2_ excess^60^ (with respect to a nominally stoichiometric composition) for efficient devices. In order to attain a suitable mid-bandgap perovskite for use in our PPP triple-junction devices, we turn to a thermally co-evaporated FAPbI_3_ first demonstrated by Borchert *et al.*^61^ in 2017, which has a suitable bandgap of around 1.5 eV for triple-junction solar cells as shown in previous models.^62^ Note, we use a set of controlled checks from Tauc plots, X-ray diffraction (XRD) and external quantum efficiency (EQE) absorption onsets to gauge our nominal perovskite compositions.^13,60^ We thermally co-evaporate FAPbI_3_ (1.53 eV) by simultaneously evaporating both FAI and PbI_2_ powders onto the rotating sample stage, as shown in Fig. 1a. Through this optimisation process, we found that the performance of resulting FAPbI_3_ perovskite solar cells is highly sensitive to the ratio between the PbI_2_ and FAI rates. We set the PbI_2_ rate constant at 0.6 Å s^−1^ whilst varying, in separate evaporation deposition runs, the FAI rate from 0.6 Å s^−1^ up to 1.5 Å s^−1^. It is noted here that when evaporating sensitive organic components, namely FAI or MAI, there is a “sticking-coefficient”, which means that the rate detected *via* the quartz crystal monitor (QCM) is much lower than what actually ends up on the substrate surface. Thus, it has been reported by us and others that excess FAI is needed to approach stoichiometric precursor ratios in the deposited films.^60,63,64^ We note that given the poor sticking characteristics of the organic source, FAI, it is difficult to accurately quantify the ratio of the FAI to PbI_2_ in the final thermally co-evaporated film, thus only the relative rates (in the Å s^−1^ of the FAI with the PbI_2_ fixed at 0.6 Å s^−1^) are reported here. Fig. 1b shows the XRD patterns of the various films deposited on glass/indium tin oxide (ITO)/(2-(3,6-dimethoxy-9*H*-carbazol-9-yl)ethyl)phosphonic acid (MeO-2PACz) substrates (representing the bottom layers of the solar cells) followed by thermal annealing on a hotplate at 150 °C for 20 min in a N_2_ glovebox environment. The three peaks of interest are the black α-FAPbI_3_ (100) phase at 14.2°, the PbI_2_ phase at 12.9°, and an unwanted yellow non-perovskite δ-FAPbI_3_ phase at 11.8°. We can see that at an FAI rate of 0.6 Å s^−1^ there is a large PbI_2_ peak as well as characteristics of both δ-FAPbI_3_ and α-FAPbI_3_ phases. For the FAI rate at 0.9 Å s^−1^, the PbI_2_ peak decreases in intensity together with the δ-FAPbI_3_, whilst the α-FAPbI_3_ peak increases. The same trend continues as we increase the rate to 1.1 Å s^−1^, as the PbI_2_ peak disappears and only the α-FAPbI_3_ peak is observable. From 1.2 Å s^−1^ the (100) peak then starts to decrease until 1.5 Å s^−1^ where there is very little detectable perovskite-related peak in the film, with the film acquiring a visible orange shade (ESI Fig. 5†). Photothermal deflection spectroscopy (PDS) measurements presented in Fig. 1c allowed us to calculate Urbach energy,^65^ a proxy for examining energetic disorder, which showed a decreasing trend from 0.9 (18.4 meV) to 1.5 Å s^−1^ (17.2 meV) with increasing rate of FAI, whereas the bandgap remained the same at 1.53 eV (Urbach energy fits and Tauc plots can be found in ESI Fig. 6†). From the top-view scanning electron microscopy (SEM) images in ESI Fig. 7d–f,† the morphological grain size increased from 170, 180, to 280 nm with increasing FAI from 0.9, 1.2 to 1.5 Å s^−1^ respectively. This was in line with a slight increase in thickness (0.9, 1.2, and 1.5 Å s^−1^ were 490, 512, and 552 nm, respectively) as seen from profilometry measurements (ESI Fig. 8†). Note, the thicknesses above vary because the total time for each run was set to be the same, dictated by the fixed PbI_2_ rate of 0.6 Å s^−1^ and fixed time, and thicknesses could be made to match if the total run time was varied. We note that evaporated FAPbI_3_ tends to have smaller grain sizes between 100 and 200 nm compared to solution-processed analogues (typically >200 nm).^66^

Single-junction FAPbI_3_ perovskite solar cells with the structure ITO/MeO-2PACz/FAPbI_3_/C60/BCP/Cu were fabricated and measured under 1-Sun conditions and AM1.5 (see Methods). Fig. 1d–g shows the *J*–*V* parameters for devices with the FAI rates of 0.6, 0.9, 1.2, and 1.5 Å s^−1^. As seen in Fig. 1d, a clear drop in open-circuit voltage (*V*_OC_) is observed as the FAI : PbI_2_ ratio increases, with the *V*_OC_ decreasing from an average of 1.08 V at 0.6 Å s^−1^ to 0.48 V at 1.5 Å s^−1^. In contrast, Fig. 1b shows that the highest *J*_SC_ values are achieved between 0.9 and 1.2 Å s^−1^, averaging around 18.8 mA cm^−2^ (0.9 Å s^−1^) and peaking at 20.9 mA cm^−2^ (1.2 Å s^−1^). We also performed a thickness comparison to show that there is no significant drop in *J*_SC_ over the thickness range of interest for our MJ solar cells (around 500–700 nm) and we only see a drop in *J*_SC_ at very high thicknesses (1750 nm) (see ESI Fig. 9†). Returning to Fig. 1d–g, it was clear that an optimum solar cell performance can be found close to 0.9 Å s^−1^, with an average efficiency of 12.7%. The general trend was that near 0.9 Å s^−1^, a slight decrease in FAI to PbI_2_ ratio leads to an increase in *V*_OC_, whereas a slight increase in FAI to PbI_2_ ratio leads to higher *J*_SC_. It is well established that a slight excess in PbI_2_ suppresses nonradiative charge carrier recombination for solution-based PSCs and leads to performance gains (especially in *V*_OC_) over stoichiometric compositions,^67–69^ as we also observe here with thermally co-evaporated samples. However, at some point for the very PbI_2_ rich samples (for example FAI <0.9 Å s^−1^ in this case), the non-perovskite δ-FAPbI_3_ phases increases, as we see in the XRD (Fig. 1b), which leads to performance losses.^70,71^ In addition, excess residual PbI_2_, in the form of amorphous phases that may not be detectable *via* XRD, can cause other photo-stability issues especially under illumination.^72–76^ On the other hand, for the very PbI_2_ deficient samples, that is, the FAI rich in this case (FAI >1.2 Å s^−1^), the *J*_SC_ drop could be attributed to the accumulation of organic species at grain boundaries which hinders charge carrier mobility and/or likely carrier injection into the charge transport layers.^69^ The average series (*R*_S_) and shunt resistance (*R*_SH_) for each rate are as follows: 0.6 Å s^−1^ (*R*_S_ = 15.8 Ω cm^2^, *R*_SH_ = 543.8 Ω cm^2^), 0.9 Å s^−1^ (*R*_S_ = 11.8 Ω cm^2^, *R*_SH_ = 503.4 Ω cm^2^), 1.2 Å s^−1^ (*R*_S_ = 24.1 Ω cm^2^, *R*_SH_ = 59.5 Ω cm^2^), and 1.5 Å s^−1^ (*R*_S_ = 88.2 Ω cm^2^, *R*_SH_ = 97.0 Ω cm^2^) (see ESI Table 2† for statistical analysis). The series resistance reaches an optimum (minimum) at 0.9 Å s^−1^ FAI rate, whereas shunt resistance continues to decrease in general with increasing FAI rate. Overall, these findings, together with our previous work on co-evaporated perovskites,^13^ show that a slight excess of PbI_2_ is important in order to achieve sufficient performance and phase stability, which is in turn consistent with other studies on thermally co-evaporated perovskites.^52,77,78^ Devices show stabilized power output over the course of 300 seconds as shown in Fig. 1h. In particular, the 0.9 Å s^−1^ device is the fastest to reach a stabilized maximum power compared to the other devices tested here. For voltage and current-density tracking, see ESI Fig. 10.†Fig. 1i shows the EQE of representative devices which match well to the *J*_SC_ measured in the *J*–*V*. The EQE integrated *J*_SC_ (EQE-*J*_SC_) trend in Fig. 1i, follows closely to that of the *J*_SC_ seen in the *J*–*V* scans (Fig. 1e). A maximum EQE-*J*_SC_ value is reached at the 1.2 Å s^−1^ sample with a value of 22.1 mA cm^−2^, which is the typical range for a standard solution-processed FAPbI_3_ solar cell.^79^ Given all the properties shown, from this point forward, we utilise the absorber with an FAI rate of 0.9 Å s^−1^ (relative to PbI_2_ rate of 0.6 Å s^−1^), which can achieve single-junction device efficiency exceeding 14%, which is competitive for an all-vapour FAPbI_3_ absorber without additives^59^ and thus as a suitable bandgap perovskite absorber for the middle subcell in our PPP triple-junction solar cell configuration.

### Monolithic PPP triple-junction solar cells – optical modelling *vs.* real devices

**Fig. 2:**
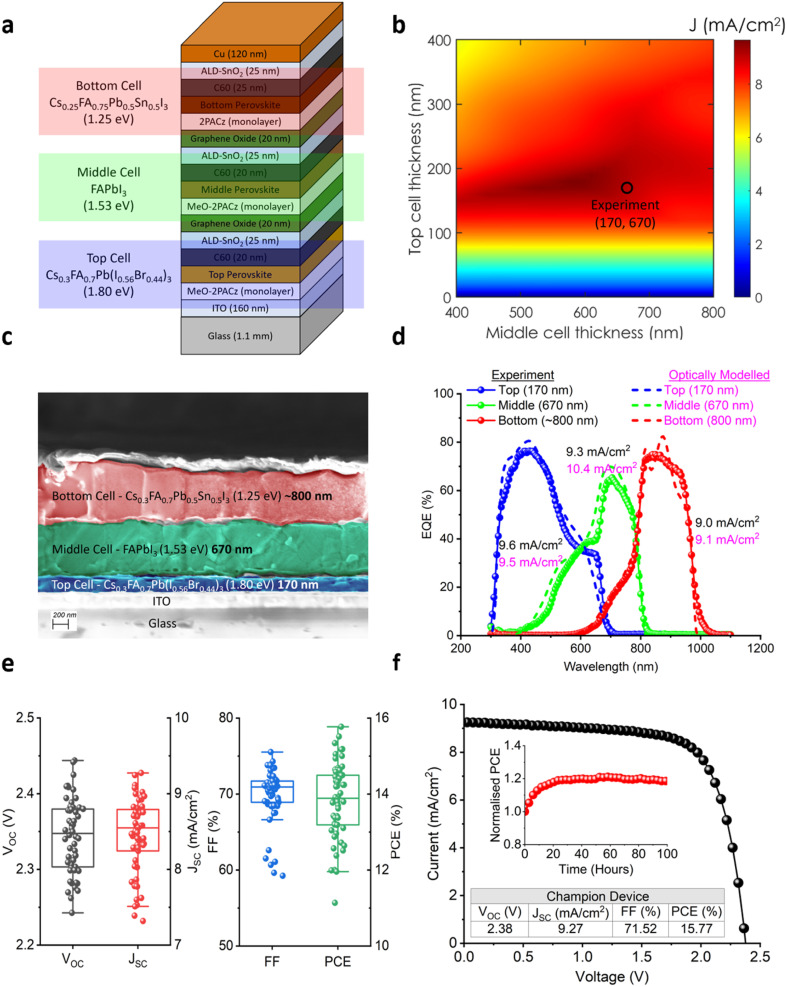
Demonstration of monolithic PPP triple-junction solar cells together with optical modelling analysis. (a) Schematic diagram of the entire device stack, (b) optically modelled *J*_SC_ heatmap of the top and middle subcell perovskite absorber thickness variation based on the stack shown in (a). Note that the optical modelling range for the top and middle perovskite absorber layers are 0–400 nm and 400–800 nm, respectively, with a fixed bottom layer thickness of 800 nm, (c) corresponding high-resolution cross-sectional SEM image, (d) EQE showing each of the three subcells including the integrated EQE-*J*_SC_ and optically modelled EQE with the same subcell absorber thicknesses. In addition, the optically modelled EQE for the maximum point (180 nm, 580 nm) in the heatmap in (c) is shown in ESI Fig. 13b,† (e) photovoltaic characteristics (*V*_OC_,*J*_SC_, FF, and PCE) for the batch, and f. Reverse *J*–*V* of the champion device (inset: 100 hours stability measurement). Note, the photovoltaic data consisted of seven substrates (8 cells each).

We performed optical simulations based on the transfer matrix method (TMM) of our proposed triple-junction device stack shown in Fig. 2a using complex refractive index data acquired from ellipsometry (see ESI Note 3 and Fig. 11†). For the top subcell we used a co-evaporated Cs_0.3_FA_0.7_Pb(I_0.56_Br_0.44_)_3_ perovskite absorber with a bandgap of 1.80 eV from our previous work,^13^ which is close to the widest possible with minimal photoinduced halide phase segregation. For the middle subcell, we used the co-evaporated FAPbI_3_ perovskite absorber (1.53 eV) as demonstrated above. Finally, for the bottom subcell, we used our solution-processed Cs_0.25_FA_0.75_Pb_0.5_Sn_0.5_I_3_ perovskite absorber (1.25 eV).^13^ For reference we show the single-junction equivalents of each type of perovskite absorber in ESI Fig. 12,† which provides a guide for performance and absorption onset. For the remaining interlayers of the stack such as the glass, ITO, fullerene C60, atomic layer deposited tin oxide (ALD-SnO_*x*_) and spin-coated graphene oxide nanoparticles (GO) we used data acquired either from public databases or measured in-house by ellipsometry (see Methods). Note, the GO layer, which we report elsewhere,^80^ is an alternative to the typical ITO or more parasitically absorbing thin Au interconnection layers typically used in MJ perovskite-based solar cells. We first performed TMM optical simulations to optimize the thicknesses of the co-evaporated top (0–400 nm) and co-evaporated middle (400–800 nm) perovskite absorber layers. The resulting *J*_SC_ heatmap, shown in Fig. 2b, plots the top absorber layer thickness (*y*-axis) against the middle absorber layer thickness (*x*-axis), with the solution-processed Pb–Sn bottom absorber fixed at 800 nm. Note, the Pb–Sn bottom absorber should be as thick as possible in terms of current collection in all-perovskite MJs, provided that sufficient charge transport can be maintained. Most work on Pb–Sn PSCs have demonstrated absorber thicknesses of less than 1 μm,^10,13,81–85^ likely due to the short carrier diffusion length and/or difficulty in achieving high quality films *via* solution-processing given solvent solubility limits and spin-coating kinetics. Lin *et al.*^86^ demonstrated thickness up to 1.2 μm in ammonium-cation-passivated Pb–Sn perovskites without loss in charge transport, thus achieving higher short-circuit current and record perovskite–perovskite tandem solar cell efficiency. The simulation results in Fig. 2b show that the maximum *J*_SC_ occurs when the top and middle absorbers, using our chosen compositions, are 180 and 580 nm, respectively. These insights provide valuable guidance for selecting the optimal layer thicknesses in the monolithic PPP triple-junction solar cells.

We fabricate a monolithic PPP triple-junction perovskite solar cell based on the stack represented in Fig. 2a with a corresponding high-resolution cross-sectional SEM image shown in Fig. 2c. In terms of the absorber thicknesses, the top thermally co-evaporated perovskite absorber, Cs_0.3_FA_0.7_Pb(I_0.56_Br_0.44_)_3_, was 170 nm (blue shade), the thermally co-evaporated middle absorber, FAPbI_3_, was 670 nm (green shade), and finally bottom subcell solution-processed perovskite, Cs_0.15_FA_0.85_Pb_0.5_Sn_0.5_I_3,_ was approximately 800 nm (red shade). From Fig. 2c, it was observed that the top and the middle subcells were very conformal driven by the thermal co-evaporation process. Referring back to Fig. 2b, the top and middle absorber thicknesses of 170 and 670 nm respectively places the efficiency reference point within the optimum current-matching region. The EQE of our champion PPP triple-junction solar cell is shown in Fig. 2d, where we can observe that the top, middle, and bottom subcells had an integrated EQE-*J*_SC_ of 9.6, 9.3, and 9.0 mA cm^−2^, respectively. Given the sensitivity of MJ devices with the illumination spectrum, we provide the spectra used in our class AAA *J*–*V* solar simulator and the EQE setups in ESI Fig. 14a and b.† As detailed in ESI Note 1,† the overall *J*_SC_ of a tandem or MJ device is constrained by the subcell with the lowest photocurrent. In our case, the limiting factor is the bottom Pb–Sn subcell, which operates at 9.0 mA cm^−2^. Note, there is reasonable agreement, although a slight difference, between the *J*_SC_ measured *via J*–*V versus* the *J*_SC_ obtained from integrated EQE measurements (EQE-*J*_SC_).^87,88^ We specifically highlight the remarkably small current variation between the subcells, with EQE-*J*_SC_ values 9.0, 9.3, and 9.6 mA cm^−2^ showing an absolute spread of less than 4% from the champion *J*_SC_ value of 9.3 mA cm^−2^ (6.7% between the highest and lowest EQE-*J*_SC_ values). In contrast, previous reports on PPP triple-junction solar cells with all solution processed perovskite layers (ESI Fig. 2†) have exhibited larger spreads between their highest and lowest integrated EQE-*J*_SC_ values of their individual subcells ranging from the largest 88.7% (ref. 29) to smallest 9.3% (ref. 32) (note, within working batches there are nevertheless some variation). Comparatively, the best III–V triple-junction solar cell made *via* metalorganic vapor phase epitaxy (another highly controllable vacuum deposition technique) has an EQE-*J*_SC_ spread of only 4.5%.^43^ The dotted lines in Fig. 2d show our optically modelled EQE, which closely aligns with the EQE of the experimental device, when using the same absorber thicknesses. This further confirms the accuracy of the thermal co-evaporation technique. Indeed, the EQE spectral behaviour and integrated EQE-*J*_SC_ of the actual experimental device matches well with that of the optical modelling, especially in the top (9.6 *vs.* 9.5 mA cm^−2^) and bottom (9.0 *vs.* 9.1 mA cm^−2^) subcells, respectively. However, for the middle subcell, there is a slight overestimation of the integrated EQE-*J*_SC_ in the optical model (10.4 mA cm^−2^) compared to the experimental device (9.3 mA cm^−2^). The fact that the modelled EQE is shifted upwards slightly is likely because in real devices there are effects such as charge generation/dissociation, non-radiative recombination, and extraction losses that are not accounted for in the optical model. Additionally, while the EQE-*J*_SC_ values of the bottom subcell are similar for both the experimental and modelled, the experimental EQE curves are smoother than the optically modelled EQE curves, where the latter exhibit more interference fringe patterns. This is likely due to the idealized smooth layers in the optical model, where interference is more pronounced. In practice, layer thickness variations and surface roughness causes increased scattering of light at more oblique angles or a more Lambertian scattering process in the experimental device *vs.* the optically modelled scenario. Hence, we emphasise that in thermally co-evaporated layers (top and middle), the features in the EQE curves in Fig. 2d match well, which is actually an argument to say that the co-evaporated layers are of better optical quality, with less thickness roughness and more accurate deposition than the solution-processed layer (bottom). In addition, we show in ESI Fig. 13a and b† the optically modelled device stack and EQE (when the top and middle absorbers are 180 and 580 nm thick, respectively) highlighting that the maximum *J*_SC_ for current matching is 9.7 mA cm^−2^. We note that, in order to develop the final monolithic PPP triple-junction solar cell structure, we performed a number of iterative steps with the development of suitable charge transport and interconnection layers (ESI Fig. 15–18 and Table 3†).

The *J*–*V* characteristics of the devices are presented in Fig. 2e. The average PV parameters across the batch were *V*_OC_ = 2.34 V, *J*_SC_ = 8.45 mA cm^−2^, FF = 69.84%, and PCE = 13.82% (reverse). The champion device achieved *V*_OC_ = 2.38 V, *J*_SC_ = 9.27 mA cm^−2^, FF = 71.52%, and PCE = 15.77% in reserve scan (forward: *V*_OC_ = 2.30 V, *J*_SC_ = 9.27 mA cm^−2^, FF = 66.58%, and PCE = 14.22%). Notably, no passivation layers were applied to any of the three subcells. Previous work has demonstrated that passivation could significantly improve *V*_OC_ and overall efficiency by reducing surface recombination,^13,89–92^ and efforts along these lines in future work will further increase *V*_OC_. We also estimate the series and shunt resistance of our champion device which were approximately 28.9 and 1.9 × 10^3^ Ω cm^2^, respectively. Additionally, the ALD-SnO_*x*_/GO recombination junctions at the top–middle and middle–bottom interfaces were not fully optimized in this instance, possibly contributing to shunt pathways.

The champion device achieved a *J*_SC_ of 9.27 mA cm^−2^, one of the highest values reported amongst monolithic PPP triple-junction solar cells in literature (ESI Fig. 3b†). Moreover, as previously mentioned, it exhibits the smallest variation in the integrated EQE-*J*_SC_ values across the individual top, middle, and bottom subcells (ESI Fig. 2b†), meaning that the current-mismatch is minimized. This is attributed to the use of the more transparent ALD-SnO_*x*_/GO interlayers^80^ than the standard ALD-SnO_*x*_/Au and, crucially, the precise thickness control enabled by the thermal co-evaporation process. Furthermore, our EQE profile has coincidentally led to the bottom subcell with the lowest current at 9.0 mA cm^−2^, and, as Boccard and Ballif^93^ pointed out, such bottom-cell-limitation appears to be the most ideal in terms of maintaining high FF values in most realistic situations. As a result, the high Fill Factor (FF) we achieve for our champion device was 71.52%, and the highest FF we achieved in this batch was 75.53%, which is reasonably competitive with existing triple-junction PPP solar cells (highest at FF = 81% (ref. 30)) (see ESI Fig. 3c†). This could also be due to the more conformal thermal co-evaporation process for the top and middle perovskite absorber layers. The *J*–*V* curve and parameters for the champion PPP triple-junction solar cell are presented in Fig. 2f, along with an inset showing the results of a 100 hours encapsulated max power stability test conducted in air with the temperature of the device held at 25 °C using a thermoelectric temperature-controlled stage (see ESI Fig. 19†). The device PCE shows an initial PCE gain (typical for p–i–n architectures^94^) up to around 60 hours and then the gradual slow decrease. At 100 hours the device was still operating above its initial starting PCE. We believe this excellent stability result is due to a combination of our solution-free thermal co-evaporation process as well as our ALD-SnO_*x*_ protective layers, especially the final layer just before the Cu contact. Thus, we have demonstrated that thermal co-evaporation can be highly versatile and thickness-controllable for MJ configurations.

### Further optimisation opportunities for monolithic PPP triple-junction solar cells

**Fig. 3:**
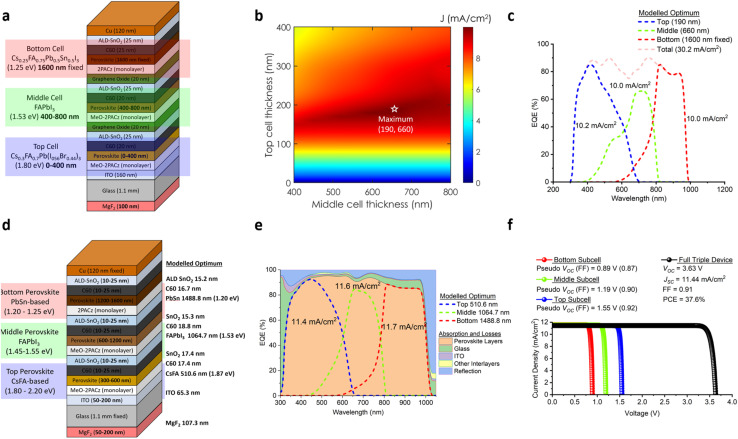
Further optimisation parameters for the demonstrated monolithic PPP triple-junction solar cell. (a) Schematic diagram of the optimal device stack from optical modelling with the addition of a front MgF_2_ anti-reflection coating and increased bottom subcell absorber thickness, (b) optically modelled *J*_SC_ heatmap of the top and middle subcell perovskite absorber thickness variation based on the stack shown in (a). Note that the optical modelling range for the top and middle perovskite absorber layers are 0–400 nm and 400–800 nm, respectively, (c) optically modelled EQE from the maximum point shown in (b). Showing each of the three subcells including the integrated EQE-*J*_SC_ and total EQE-*J*_SC_, (d) further optimized device stack with a thickness sweep of all active layers with the ranges shown inside the stack and the optimum values on the right in bold, (e) optically modelled EQE from the stack shown in (d). Showing each of the three subcells including the integrated EQE-*J*_SC_ and breakdown of the absorption and losses, and (f) optoelectronically simulated *J*–*V* curves of the monolithic PPP triple-junction solar cell including each of the subcell contributions, where the *J*_SC_ is based on the values modelled in (e). The simulated PV parameters of the full triple-junction device are also shown.

Finally, we use optical modelling to identify further areas for performance gain (Fig. 3a) based on our three perovskite absorber layers. We first added a 100 nm MgF_2_ antireflection coating (ARC) at the front side of the glass substrate to improve light coupling into the active layers of our device. Secondly, we doubled the Pb–Sn bottom subcell thickness to 1600 nm to improve the long-wavelength absorption. Fig. 3b shows the results of this simulation in terms of a thickness optimisation heatmap for the top and middle perovskite absorbers of the stack shown in Fig. 3a. Fig. 3c shows the optimum EQE (corresponding to the maximum point in Fig. 3b where the top and bottom absorber thicknesses are 190 and 660 nm respectively) with current matching now slightly improved to 10.0 mA cm^−2^. With the MgF_2_ ARC and thicker Pb–Sn bottom subcell, the total current available in the new simulation increased from 29.1 mA cm^−2^ (sum of EQE-*J*_SC_ values in ESI Fig. 14b†) to 30.2 mA cm^−2^ (sum of EQE-*J*_SC_ values in Fig. 3c). This in turn has shifted the optimum thicknesses of the top and middle absorber pairs from 180 and 580 nm (Fig. 2b) to 190 to 660 nm (Fig. 3c), respectively.

We further improved this device stack by optimizing the thicknesses of the interlayers. As shown in Fig. 3d, we established realistic thickness parameters for each interlayer, including MgF_2_, ITO, C60, ALD-SnO_*x*_, and our three perovskite absorber layers. The aim was to minimize parasitic absorption and improve light coupling between the interlayers to optimize optical transparency to the perovskite absorbers. Furthermore, we sweep through suitable bandgap ranges for the top, middle, and bottom perovskites to find the optimal bandgap combination. The optimal thicknesses and bandgaps of each perovskite layer as well as the thicknesses for each interlayer modelled are highlighted in bold on the right side of Fig. 3d. The top, middle, and bottom perovskite layers had optimum bandgaps of 1.87, 1.53 and 1.20 eV and thicknesses of 510.6, 1064.7, and 1488.8 nm, respectively (see ESI Fig. 20† for the simulated complex refractive index data). As a result of this optimization, Fig. 3e shows the optimal EQE based on these tuned layer thicknesses. Here we observe an improved *J*_SC_ matching of 11.4 mA cm^−2^ (top subcell limited), representing a significant improvement over our earlier stacks (ESI Fig. 14† with 9.7 mA cm^−2^ and Fig. 3c with 10.0 mA cm^−2^). This improvement is firstly due to the increased total absorption range with a lower bottom subcell bandgap of 1.20 eV and secondly, largely due to the significant reduction in the parasitic absorption of the interlayers, especially in the C60 and ITO. It should be noted here that this result comes from an optical model that does not account for changes in material properties with thickness. For example, in this model, we assume that reducing the ITO thickness from 160 nm to 65.3 nm will maintain comparable sheet resistance. In reality, this could be difficult given the relationship between resistivity, transmittance, doping, and film thickness in transparent conducting oxides^95,96^ what may make this challenging. The enhanced light management and improved light absorption and coupling within the device itself also play a crucial role in the performance gains. In Fig. 3e we can see the various absorption components and losses. The glass absorbs more highly in the shorter wavelengths typically below 350 nm and then again towards the red and infrared regions. The ITO absorption losses also mainly occur at the shorter wavelength region, whereas the interlayer losses have a more even distribution in terms of the wavelength range. The perovskite layers contribute to the bulk of the absorption resulting in a total current density of 34.7 mA cm^−2^. Finally, the inevitable reflection losses in this case are minimized through optimizing for the thicknesses of the various interlayers, especially the front ARC. Note, we only show one ARC layer here, although more than one ARC^97^ can be used, however this comes with diminishing returns.

By allowing bandgaps and thicknesses of the perovskite absorbers to vary more widely together with the careful tuning of the thickness of the other layers, the practical efficiency limit for this new stack is thus much higher. We run a simple simulation based on the modelling to assess this practical efficiency limit. A simulated *J*–*V* curve including those of the subcells are shown in Fig. 3e. Here we see that the pseudo-*V*_OC_ that can be achieved by the top, middle, and bottom subcells in the stack are 1.55, 1.19, and 0.89 V, respectively. Together with a *J*_SC_ matching of 11.4 mA cm^−2^ based on our perovskite absorbers with bandgaps (1.87, 1.53, and 1.20 eV) and estimated FF of 0.91 (see methods) we can therefore estimate an optimistic, but close to achievable, device efficiency of ∼37.6%.

The improvements that have to be made *versus* existing monolithic PPP triple-junction solar cells include: (1) improving the *V*_OC_ of the high-bandgap perovskite materials without suffering from the effects of halide segregation,^32,36,38,40,98^ (2) increasing the bottom subcell Pb–Sn perovskite absorber thickness without loss in current extraction (where a thermally co-evaporated Pb–Sn perovskite could be made as thick as required without precursor concentration limitations^99^), (3) find intermediate layers that are thinner and more transparent, yet equally functional, and finally (4) better optoelectronic simulations to assist in fabrication of real devices.

## Conclusion

In this work, we first demonstrated the successful fabrication of thermally co-evaporated FAPbI_3_ perovskite films and solar cells using 2-sources, FAI and PbI_2_. This technique is especially useful compared to conventional solution processing, as it allows conformal deposition that is solution-free with excellent thickness control. We show that the ratio of the evaporation rates between the FAI and PbI_2_ has a large effect on the properties of the final FAPbI_3_ perovskite. Specifically, we found that a PbI_2_ rich composition, which in our case means a lower FAI (0.9 Å s^−1^) to PbI_2_ (0.6 Å s^−1^) evaporation rate, resulted in the most efficient working devices overall with higher *V*_OC_ and FF. However, a slightly more stoichiometric FAI to PbI_2_ ratio, where the FAI and PbI_2_ rates were 1.2 Å s^−1^ to PbI_2_ 0.6 Å s^−1^, respectively, yielded better *J*_SC_ at the expense of lower *V*_OC_ and FF. We then successfully demonstrated a working 2-terminal monolithic PPP triple-junction solar cell with *V*_OC_ of 2.38 V, *J*_SC_ of 9.27 mA cm^−2^, FF of 71.52%, PCE of 15.77% using thermal co-evaporation for the deposition of the 4-source Cs_0.3_FA_0.7_Pb(I_0.56_Br_0.44_)_3_ top cell and our optimized 2-source FAPbI_3_ middle cell absorbers. The integrated EQE-*J*_SC_ for the top, middle, and bottom subcells were 9.6, 9.3, and 9.0 mA cm^−2^ respectively, which is one of the most effectively current-matched for monolithic triple-junction solar cells in literature. These results are owing to our effective optical simulations and the excellent versatility of the thermal co-evaporation process, whereby the bandgap and thickness of the perovskite absorber can be controlled effectively. Furthermore, because the thermal co-evaporation technique is solution-free and in vacuum, subsequent layer deposition of the perovskite absorber avoids damaging, or at least, reduces the damage to underlying layers. Our work opens further doors to the vacuum-deposition thermal co-evaporation technique for perovskite research and commercialisation.

## Methods

### Materials

All materials were used as received without further purification. FAI (99.99%) was purchased from Greatcell Solar Materials (Australia). MeO-2PACz (>98.0%), 2PACz (98.0%), PbI_2_ (99.99% trace metal basis) and PbBr_2_ (99.99% trace metal basis) were purchased from Tokyo Chemical Industry Co. (Japan). Cesium bromide (99.999% trace metals basis), tin iodide (beads, 99.99%), tin flouride (99%), cesium iodide (99.999% trace metals basis), *N*,*N*-dimethylformamide (anhydrous, 99.8%), dimethyl sulfoxide (anhydrous >99.9%), PTAA, toluene (anhydrous, 99.8%), ethanol (anhydrous, 99.5%) were purchased from Sigma-Aldrich (Global). BCP (>99.5% sublimed) and PEDOT : PSS was purchased from Ossila. C60 (99.99%) was purchased from Creaphys (subsidiary of MBraun) GmBH (Germany). Single-layer graphene oxide dispersion in water was purchased from Graphene Supermarket (USA). Patterned ITO (10–15 ohm per sq.) 25.4 × 25.4 × 1.1 mm substrates were purchased from Kintec (Hong Kong). TDMASn precursor was purchased from Strem Chemicals (USA).

### Device fabrication

#### Substrate preparation

ITO substrates (Kintec) were cleaned in a sonication bath in Hellmanex solution, DI water, acetone and isopropanol (15 min each). The cleaned substrates were transferred to a UV-ozone chamber (UVC1014, NanoBioAnalytics) for another 15 minutes post-treatment.

#### Wide-bandgap top perovskite and solar cell fabrication

120 μl of MeO-2PACz (0.4 mg ml^−1^) in anhydrous ethanol was dropped on the cleaned ITO substrate and spincoated at 4000 r.p.m. for 30 seconds in a glovebox with an integrated spin-coater followed by post-annealing at 100 °C for 10 min. The MeO-2PACz substrates were transferred to a PEROevap (CreaPhys/Mbraun) chamber inside a N_2_-filled glovebox for perovskite evaporation. The chamber was pumped down to below 2.0 × 10^−6^ mbar. During the evaporation, the substrates stage was kept at 18 °C temperature, while the chamber walls were kept at −15 °C temperature. The precursor deposition rates for the four sources were 0.9 Å s^−1^ for FAI, 0.6 Å s^−1^ for PbI_2_, 0.1 Å s^−1^ for PbBr_2_, and 0.1 Å s^−1^ for CsBr. The PbI_2_ powders were placed in an alumina crucible whereas the FAI powder, being a more sensitive organic molecule, was placed in a crucible of a special “ultra-low temperature” source fixture designed for controlled heating. After the perovskite evaporation, the samples were post annealed at 150 °C or 170 °C for 20 minutes to form the Cs_0.3_FA_0.7_Pb(I_0.56_Br_0.44_)_3_. To complete the single-junction perovskite solar cells, the samples were transferred back into the perovskite evaporator for C60 (25 nm), BCP (8 nm) and Cu (120 nm) deposition.

#### Mid-bandgap middle cell perovskite and single-junction solar cell fabrication

120 μl of MeO-2PACz (0.4 mg ml^−1^) in anhydrous ethanol was dropped on the cleaned ITO substrate and spincoated at 4000 r.p.m. for 30 seconds in a glovebox with integrated spin-coater, followed by post-annealing at 100 °C for 10 min. The MeO-2PACz substrates were transferred to a PEROevap (CreaPhys/Mbraun) chamber inside a N_2_-filled glovebox for perovskite evaporation. The chamber was pumped down to below 2.0 × 10^−6^ mbar. During the evaporation, the substrates stage was kept at 18 °C temperature, while the chamber wall was at −15 °C temperature. The precursor deposition rates for the two sources were 0.9 Å s^−1^ for FAI and 0.6 for PbI_2_. The PbI_2_ powders were placed in an alumina crucible whereas the FAI powder, being a more sensitive organic molecule, was placed in a crucible of a special “ultra-low temperature” source fixture designed for controlled heating. After the perovskite evaporation, the samples were post annealed at 150 °C for 20 minutes to form the FAPbI_3_ perovskite. To complete the single-junction perovskite solar cells, the samples were transferred back into the perovskite evaporator for C60 (25 nm), BCP (8 nm) and Cu (120 nm) deposition.

#### Low-bandgap bottom perovskite and solar cell single-junction solar cell fabrication

120 μl of 2PACz (0.3 mg ml^−1^) in anhydrous ethanol was dropped on the cleaned ITO substrate and spincoated at 3000 r.p.m. for 30 seconds in a glovebox with integrated spin-coater, followed by post-annealing at 100 °C for 10 min. Anhydrous ethanol was then dropped on the film and again spincoated at 3000 r.p.m. for 30 seconds to remove the excess 2PACz, followed by annealing at 100 °C for 2 minutes. To make the perovskite solution, the precursors were mixed in 1 × 4 ml vial in the following order, SnF_2_ (0.1 M), SnI_2_ (1 M), CsI (0.5 M), PbI_2_ (1 M) and FAI (1.5 M) to achieve 2 M concentration in DMF : DMSO (4 : 1) for the composition of Cs_0.25_FA_0.75_Pb_0.5_Sn_0.5_I_3_. The Pb–Sn perovskite solution was prepared in a N_2_-filled glovebox (H_2_O and O_2_ below 1 ppm) and stirred for at least 3 hours before use. 120 μl perovskite solution was spread on the 2PACz/ITO substrate and spun at 5000 r.p.m. for 40 s for with N_2_ gas quenching at 25 s into the process (some devices at 4000 rpm and 30 s N_2_ gas quenching total). The samples were moved to a hotplate for post-annealing at 120 °C for up to 10 min.

#### Monolithic triple-junction PPP solar cell fabrication

This method was based on the triple-junction shown in the main text. The configuration of the PPP triple-junction was Glass/ITO/MeO-2PACz/1.83 eV Cs_0.3_FA_0.7_Pb(I_0.56_Br_0.44_)_3_ perovskite/C60/SnO_*x*_/GO/MeO-2PACz/1.53 eV FAPbI_3_ perovskite/C60/SnO_*x*_/GO/2PACz/1.25 eV Cs_0.25_FA_0.75_Pb_0.5_Sn_0.5_I_3_ perovskite/C60/SnO_*x*_/Cu. The perovskite, MeO-2PACz, 2PACz, C60, and Cu were deposited in the same fashion as described in the above sections. The use of 2PACz as the hole transport layer for the bottom solution-processed Cs_0.25_FA_0.75_Pb_0.5_Sn_0.5_I_3_ recipe was based on previous works^85,100^ showing improved performance when PEDOT : PSS was replaced by 2PACz. For the thermally co-evaporated top and middle cells, MeO-2PACz was used based on our previous works on thermally co-evaporated perovskites,^13,77^ where we empirically found improved performance compared to 2PACz. The SnO_*x*_ was deposited *via* atomic layer deposition (Picosun) using (TDMA)Sn and deionised water H_2_O as precursors. The base chamber pressure was 10 mbar. The recipe involved alternating cycles of the TDMASn pulse/N_2_ purge/H_2_O pulse/N_2_ purge time were 0.8 s, 15 s, 0.2 s and 15 s for 250 cycles (target thickness: 25 nm SnO_*x*_). The substrate plate temperature was kept constant at 100 °C. The temperature of the source bottle and line/neck were kept at 75 and 100 °C respectively. A boost system for the N_2_ carrier gas was also used to increase the vapour of TDMASn. GO was spin-coated at a volume of 100 μl and concentration of 0.35 mg ml^−1^ H_2_O onto the substrates in a fumehood in air at 4000 rpm for 30 seconds and then placed on a hotplate at 100 °C for 10 min.

Note, the annealing temperature of various stacks needed to be taken into account, as annealing of a subsequent stack that were deposited later would mean annealing the entire MJ stack at that temperature, which may not be ideal for low-temperature perovskites or other more sensitive layers, such as the organic transport layers. Hence, we annealed each absorber stack section staggered at a decreasing temperature after each deposition step, starting from the highest temperature, 170 °C for the top cell for the shortest duration, to the middle 150 °C, and then the bottom cell 100 °C for the longest duration. This would avoid or at least reduce the likelihood of destroying underlying layer stacks.

### Optical modelling method

Optical modelling simulations of the device stacks were performed using a general transfer matrix model (TMM) using the spectral refractive index data of the layers. The complex refractive indices of the perovskite films were obtained through ellipsometry and were used as inputs into the TMM program. For the more universal layers, such as the ITO, C60, SnO_*x*_, and Cu refractive indices were taken from online databases (for example, https://refractiveindex.com/). For more information refer to ESI Note 3.†

For the PCE and *J*–*V* analysis we developed a simple model based on the previously calculated short-circuit currents for the optimal case from our optical modelling above. We then used eqn (1) (ref. 101) based on the Kirchoff radiation law and Planck generalized law to calculate the recombination current for each subcell. The presence of the value “2” before the integrals comes from a geometrical factor.^2^ These recombination currents were combined with the short-circuit currents in order to then calculate the pseudo open-circuit voltages for each of the subcells from eqn (2). The absolute pseudo open-circuit voltages were obtained with the recombination currents evaluated at *V* = 0. The use of eqn (2) for the calculation of *V*_OC_ is equivalent to evaluate the *J*–*V* curve of each subcell at the voltage where the current *J* reaches the value 0. By looking at eqn (3), *V*_OC_ would be obtained by the operation *V* (*J* = 0). The 1/3 and 2/3 factors were used in the *V*_OC_ formulas for the middle and bottom subcell, due to the fact that both respectively lose 1/3 and 2/3 of the light because of the absorption of the upper layers (see ESI Note 1†). Using the combination of the *J*–*V* curves of each of the three subcells, the final *J*–*V* curve and PV parameters for the triple-junction device could then be determined. In order to obtain the *J*–*V* curve for the entire device we plotted eqn (3) depending on the voltage for each subcell and considering that the subcells are connected in series, they must verify the current matching condition (see eqn (2) from ESI Note 1†). The *V*_OC_ of the complete device is obtained again by evaluating the voltage, *V* at which the current *J* attains the value 0. The pseudo fill factors for each subcell and the complete device were then calculated likewise using eqn (4) that involves *P*_max_, the maximum power at which the solar cell could theoretically operate. Then, *P*_max_ is the maximum value of the product of the current, *J* and the voltage, *V* involved in the *J*–*V* curves. Finally, using all these parameters, we were able to calculate the PCE of the complete device.1
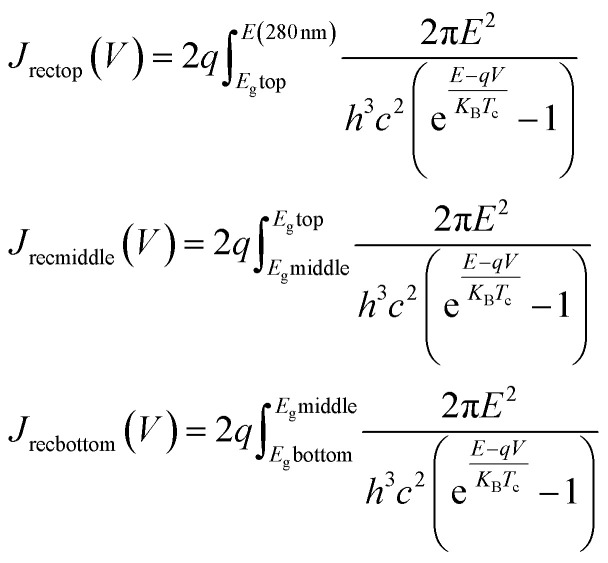
2
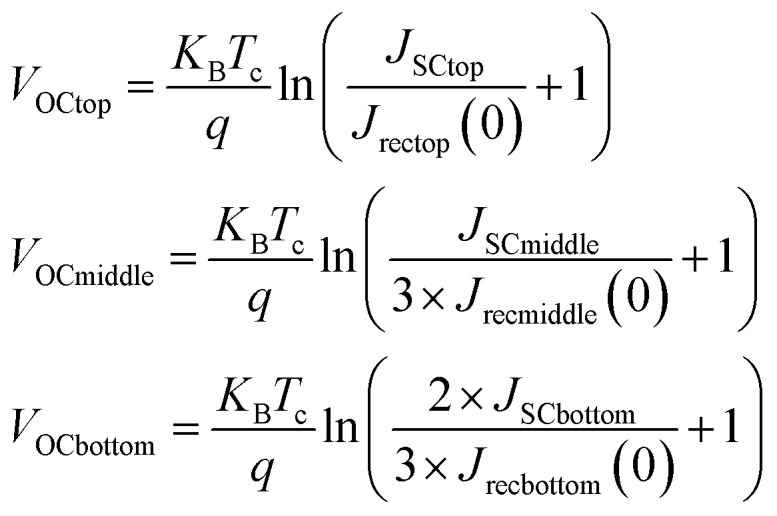

3 *J* = *J*_SC_ − *J*_rec_(*V*)

4
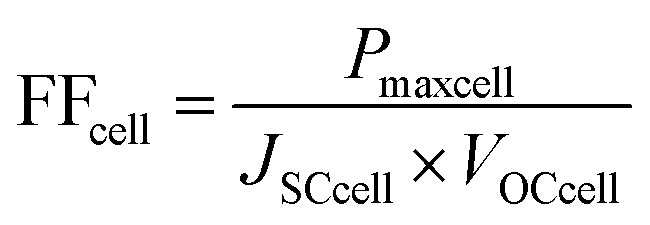


### Characterisation of solar cells

#### *J*–*V*

The current density–voltage (*J*–*V*) characteristics were measured using a custom-built Cicci measurement setup. *J*–*V* curves were measured in air with a forward/reverse scan rate of 200 mV s^−1^. All solar cells were measured under the standard 1-Sun AM 1.5G spectrum using a Sunbrick Base-UV large area AAA LED solar simulator (G2V), with a spectral mismatch of <5%. The system was calibrated separately using both a silicon KG5 filter reference cell (RERA Solutions, model number: RK5N3199) and an Avantes (AvaSpec-ULS2048CL-EVO-FCPC) spectroradiometer. Computer numerical controlled (CNC) metal masks with a circular aperture area of 11.8 mm^2^ were used. For the stability tracking measurements, the solar cells were fixed at the MPP voltage (as determined from the *J*–*V* sweeps). The devices were mounted in a custom designed holder (Cicci) with a thermoelectric cell base to maintain the temperature at 25 °C during the process. Most of the devices were encapsulated (UV-curable epoxy) in N_2_-filled glovebox before testing. All devices were measured in an ambient air environment unless specified.

#### EQE

A Bentham PVE300 system equipped with dual lamps of xenon and quartz-tungsten halogen lamps was used for measuring the EQE of the devices. A Newport silicon reference cell was used to measure the signal response for calibration. A spectral range from 300 up to 1100 nm was used with a step size of 5 nm. Thorlabs LED biasing sources were used for measuring the triple-junction perovskite solar cells. For measuring the top subcell, a 730 nm LED lamp was used. For the bottom subcell a 365 nm LED lamp was used. For the middle subcell a combination of both a 365 nm and 940 nm LED lamps were used. During all measurements, a transformer module preamplifier (Bentham S400 474) with a frequency of 300 Hz was employed for the silicon calibrated cell and perovskite solar cells.

### Materials characterisation

#### XRD

X-ray diffraction patterns were obtained using a Bruker D8 ADVANCE system equipped with a Cu K-alpha X-ray source (1.54 Å) at an operation voltage of 40 kV. The samples were kept in a nitrogen glovebox before transferring to the XRD system which is in ambient air atmosphere during the measurement. The scan range for 2*θ* was set from 5 to 55°, with a step size of 0.01° and a dwell time of 0.15 s per step. The samples were measured on patterned ITO glass substrates.

#### Profilometry

Profilometry was conducted with a Bruker DektakXT stylus profilometer. Up to six measurements were taken per substrate and an average was taken. We estimate an accuracy of ±5 nm for our thermally co-evaporated perovskite films.

#### PDS and Urbach energy fit

Photothermal deflection spectroscopy measurements were performed at room temperature, utilising the transverse configuration. The pump beam, created by a broadband quartz–tungsten–halogen (QTH) lamp filtered with a grating monochromator, was modulated with a mechanical chopper at 13 Hz and transferred to the sample area with a fiber to limit vibrations. The probe beam from a diode laser (670 nm) was passed parallel to the active layer, crossing the area excited with the pump beam. Absorption-induced alternating temperature gradient at the sample surface caused synchronous deviations of the probe beam optical path, measured with a quadrant photodiode coupled to a lock-in amplifier (Stanford Research Systems SR830). The samples were immersed in a thermooptic liquid (3 M™ Fluorinert™ FC-72) to enhance the effect. The Urbach energies (*E*_U_) were obtained by fitting the photon-energy-dependent absorbance data *A*(*E*) below the saturation level to the formula:
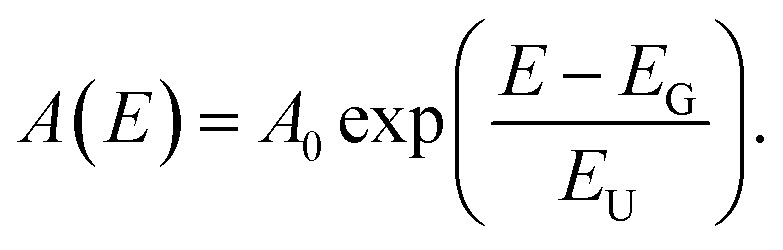


#### Cross-sectional SEM

For the FAPbI_3_ samples, SEM images were taken by a FEI Helios scanning electron microscope using the ETD detector for secondary and back-scattered electron detection. Images were taken at acceleration voltages of 2.0 kV and low-enough beam currents of 0.20 nA to ensure minimal beam damage.

For the triple-junction sample, cross-sectional SEM images were taken by a Zeiss Gemini scanning electron microscope using the Inlens detector for secondary and back-scattered electron detection. The samples were mechanically cleaved under N_2_. Images were taken at acceleration voltages of 3.0 kV and 30 μm aperture beam size to ensure minimal beam damage.

## Data availability

Data from the manuscript has been made available at the University of Cambridge's repository Apollo at https://doi.org/10.17863/CAM.115104.

## Author contributions

T. C. J.-Y. and S. D. S. conceived the idea for this project. T. C. J. Y., T. K., Y. L., fabricated and tested the thermally co-evaporated films. T. C. J. Y., T. K., M. F., Y. L., Y. H. C., Y. H. fabricated the PPP triple-junction devices. T. K., Y. H. C. fabricated and measured the single-junction Cs_0.3_FA_0.7_Pb(I_0.56_Br_0.44_)_3_ top cells. T. C. J. Y., fabricated and measured the single-junction FAPbI_3_ middle cells. M. F., Y. H. fabricated and measured the single-junction Cs_0.25_FA_0.75_Pb_0.5_Sn_0.5_I_3_ bottom cells. T. C. J. Y., T. K., performed *J*–*V*, *J*–*V* stability, and EQE of the PPP triple-junction devices. G. V., L. R., and A. J. S. performed the TMM optoelectronic modelling studies. T. C. J. Y. and Y. L. deposited FAPbI_3_ films and performed the XRD. S. J. Z. performed the PDS measurements and analysis. L. F. P. developed the encapsulation of devices, assisted in ALD optimisation and deposition, and SEM image editing. J. F. O., T. C. J. Y., Y. L. performed the SEM measurements and image analysis. Z. Y. O. performed ellipsometry, UV-vis, and AFM of the perovskite and related sample films. Y. H. C. developed the top subcell, tandem structure and earlier recipes. D. G. performed the TRPL measurements and analysis. B. R. and X. L. managed the *J*–*V*, *J*–*V* stability, and EQE setup and characterisation and helped with device analysis. C. D., S. C. P., M. A., and S. D. S. supervised the work. T. C. J. Y. wrote the first draft of the work. S. D. S. secured funding. All the authors contributed to the discussion of the results and the final manuscript preparation.

## Conflicts of interest

The authors declare the following competing financial interest(s): S. D. S. is a co-founder of Swift Solar.

## Supplementary Material

EL-001-D4EL00012A-s001

## References

[cit1] Best Research-Cell Efficiency Chart, https://www.nrel.gov/pv/cell-efficiency.html

[cit2] Shockley W., Queisser H. J. (1961). Detailed balance limit of efficiency of p–n junction solar cells. J. Appl. Phys..

[cit3] Guillemoles J.-F., Kirchartz T., Cahen D., Rau U. (2019). Guide for the perplexed to the Shockley–Queisser model for solar cells. Nat. Photonics.

[cit4] Jiang F. (2016). *et al.*, A two-terminal perovskite/perovskite tandem solar cell. J. Mater. Chem. A.

[cit5] Eperon G. E. (2016). *et al.*, Perovskite-perovskite tandem photovoltaics with optimized band gaps. Science.

[cit6] Forgács D. (2017). *et al.*, Efficient Monolithic Perovskite/Perovskite Tandem Solar Cells. Adv. Energy Mater..

[cit7] Sheng R. (2017). *et al.*, Monolithic Wide Band Gap Perovskite/Perovskite Tandem Solar Cells with Organic Recombination Layers. J. Phys. Chem. C.

[cit8] Lin R. (2019). *et al.*, Monolithic all-perovskite tandem solar cells with 24.8% efficiency exploiting comproportionation to suppress Sn(ii) oxidation in precursor ink. Nat. Energy.

[cit9] Palmstrom A. F. (2019). *et al.*, Enabling Flexible All-Perovskite Tandem Solar Cells. Joule.

[cit10] Tong J. (2019). *et al.*, Carrier lifetimes of >1 μs in Sn-Pb perovskites enable efficient all-perovskite tandem solar cells. Science.

[cit11] Prasanna R. (2019). *et al.*, Design of low bandgap tin–lead halide perovskite solar cells to achieve thermal, atmospheric and operational stability. Nat. Energy.

[cit12] Yu Z. (2020). *et al.*, Simplified interconnection structure based on C60/SnO2-x for all-perovskite tandem solar cells. Nat. Energy.

[cit13] Chiang Y.-H. (2023). *et al.*, Vacuum-Deposited Wide-Bandgap Perovskite for All-Perovskite Tandem Solar Cells. ACS Energy Lett..

[cit14] Chen H. (2023). *et al.*, Regulating surface potential maximizes voltage in all-perovskite tandems. Nature.

[cit15] Lin R. (2023). *et al.*, All-perovskite tandem solar cells with 3D/3D bilayer perovskite heterojunction. Nature.

[cit16] Mailoa J. P. (2015). *et al.*, A 2-terminal perovskite/silicon multijunction solar cell enabled by a silicon tunnel junction. Appl. Phys. Lett..

[cit17] Bush K. A. (2017). *et al.*, 23.6%-Efficient Monolithic Perovskite/Silicon Tandem Solar Cells With Improved Stability. Nat. Energy.

[cit18] Shen H. (2018). *et al.*, In situ recombination junction between p-Si and TiO _2_ enables high-efficiency monolithic perovskite/Si tandem cells. Sci. Adv..

[cit19] Sahli F. (2018). *et al.*, Fully textured monolithic perovskite/silicon tandem solar cells with 25.2% power conversion efficiency. Nat. Mater..

[cit20] Mazzarella L. (2019). *et al.*, Infrared Light Management Using a Nanocrystalline Silicon Oxide Interlayer in Monolithic Perovskite/Silicon Heterojunction Tandem Solar Cells with Efficiency above 25%. Adv. Energy Mater..

[cit21] Zheng J. (2018). *et al.*, Large area efficient interface layer free monolithic perovskite/homo-junction-silicon tandem solar cell with over 20% efficiency. Energy Environ. Sci..

[cit22] Al-Ashouri A. (2020). *et al.*, Monolithic perovskite/silicon tandem solar cell with >29% efficiency by enhanced hole extraction. Science.

[cit23] Chen B. (2020). *et al.*, Blade-Coated Perovskites on Textured Silicon for 26% -Efficient Monolithic Perovskite/Silicon Tandem Solar Cells Blade-Coated Perovskites on Textured Silicon for 26% -Efficient Monolithic Perovskite/Silicon Tandem Solar Cells. Joule.

[cit24] Hou Y. (2020). *et al.*, Efficient Tandem Solar Cells with Solution-Processed Perovskite on Textured Crystalline Silicon. Science.

[cit25] Schulze P. S. C. (2020). *et al.*, 25.1% High-Efficiency Monolithic Perovskite Silicon Tandem Solar Cell with a High Bandgap Perovskite Absorber. Sol. RRL.

[cit26] De Bastiani M. (2021). *et al.*, Efficient bifacial monolithic perovskite/silicon tandem solar cells *via* bandgap engineering. Nat. Energy.

[cit27] Tockhorn P. (2022). *et al.*, Nano-optical designs for high-efficiency monolithic perovskite–silicon tandem solar cells. Nat. Nanotechnol..

[cit28] Chin X. Y. (2023). *et al.*, Interface passivation for 31.25%-efficient perovskite/silicon tandem solar cells. Science.

[cit29] McMeekin D. P. (2019). *et al.*, Solution-Processed All-Perovskite Multi-junction Solar Cells. Joule.

[cit30] Wang J. (2020). *et al.*, 16.8% Monolithic all-perovskite triple-junction solar cells *via* a universal two-step solution process. Nat. Commun..

[cit31] Xiao K. (2020). *et al.*, Solution-Processed Monolithic All-Perovskite Triple-Junction Solar Cells with Efficiency Exceeding 20%. ACS Energy Lett..

[cit32] Wang Z. (2023). *et al.*, Suppressed phase segregation for triple-junction perovskite solar cells. Nature.

[cit33] Werner J. (2018). *et al.*, Perovskite/Perovskite/Silicon Monolithic Triple-Junction Solar Cells with a Fully Textured Design. ACS Energy Lett..

[cit34] Zheng J. (2022). *et al.*, Monolithic Perovskite–Perovskite–Silicon Triple-Junction Tandem Solar Cell with an Efficiency of over 20%. ACS Energy Lett..

[cit35] Heydarian M. (2023). *et al.*, Monolithic Two-Terminal Perovskite/Perovskite/Silicon Triple-Junction Solar Cells with Open Circuit Voltage >2.8 V. ACS Energy Lett..

[cit36] Xu F. (2024). *et al.*, Monolithic perovskite/perovskite/silicon triple-junction solar cells with cation double displacement enabled 2.0 eV perovskites. Joule.

[cit37] Choi Y. J., Lim S. Y., Park J. H., Ji S. G., Kim J. Y. (2023). Atomic Layer Deposition-Free Monolithic Perovskite/Perovskite/Silicon Triple-Junction Solar Cells. ACS Energy Lett..

[cit38] Li F. (2024). *et al.*, Highly Efficient Monolithic Perovskite/Perovskite/Silicon Triple-Junction Solar Cells. Adv. Mater..

[cit39] Hu H. (2024). *et al.*, Triple-junction perovskite–perovskite–silicon solar cells with power conversion efficiency of 24.4%. Energy Environ. Sci..

[cit40] Liu S. (2024). *et al.*, Triple-junction solar cells with cyanate in ultrawide-bandgap perovskites. Nature.

[cit41] Isikgor F. H. (2022). *et al.*, Monolithic Perovskite–Perovskite–Organic Triple-Junction Solar Cells with a Voltage Output Exceeding 3 V. ACS Energy Lett..

[cit42] Hörantner M. T. (2017). *et al.*, The Potential of Multijunction Perovskite Solar Cells. ACS Energy Lett..

[cit43] France R. M. (2022). *et al.*, Triple-junction solar cells with 39.5% terrestrial and 34.2% space efficiency enabled by thick quantum well superlattices. Joule.

[cit44] Green M. A. (2024). *et al.*, Solar cell efficiency tables (Version 63). Prog. Photovolt.: Res. Appl..

[cit45] Xu F. (2024). *et al.*, Four-Terminal Perovskite/Perovskite/Silicon Triple-Junction Tandem Solar Cells with over 30% Power Conversion Efficiency. ACS Energy Lett..

[cit46] Roose B. (2024). *et al.*, Electrochemical Impedance Spectroscopy of All-Perovskite Tandem Solar Cells. ACS Energy Lett..

[cit47] Ávila J., Momblona C., Boix P. P., Sessolo M., Bolink H. J. (2017). Vapor-Deposited Perovskites: The Route to High-Performance Solar Cell Production?. Joule.

[cit48] Vaynzof Y. (2020). The Future of Perovskite Photovoltaics — Thermal Evaporation or Solution Processing?. Adv. Energy Mater..

[cit49] Kosasih F. U., Erdenebileg E., Mathews N., Mhaisalkar S. G., Bruno A. (2022). Thermal evaporation and hybrid deposition of perovskite solar cells and mini-modules. Joule.

[cit50] Abzieher T. (2024). *et al.*, Vapor phase deposition of perovskite photovoltaics: short track to commercialization?. Energy Environ. Sci..

[cit51] Longo G. (2018). *et al.*, Fully Vacuum-Processed Wide Band Gap Mixed-Halide Perovskite Solar Cells. ACS Energy Lett..

[cit52] Roß M. (2021). *et al.*, Co-Evaporated Formamidinium Lead Iodide Based Perovskites with 1000 h Constant Stability for Fully Textured Monolithic Perovskite/Silicon Tandem Solar Cells. Adv. Energy Mater..

[cit53] Lohmann K. B. (2022). *et al.*, Solvent-Free Method for Defect Reduction and Improved Performance of p-i-n Vapor-Deposited Perovskite Solar Cells. ACS Energy Lett..

[cit54] Susic I., Gil-Escrig L., Palazon F., Sessolo M., Bolink H. J. (2022). Quadruple-Cation Wide-Bandgap Perovskite Solar Cells with Enhanced Thermal Stability Enabled by Vacuum Deposition. ACS Energy Lett..

[cit55] Liu M., Johnston M. B., Snaith H. J. (2013). Efficient planar heterojunction perovskite solar cells by vapour deposition. Nature.

[cit56] Momblona C. (2016). *et al.*, Efficient vacuum deposited p-i-n and n-i-p perovskite solar cells employing doped charge transport layers. Energy Environ. Sci..

[cit57] Ávila J. (2018). *et al.*, High voltage vacuum-deposited CH _3_ NH _3_ PbI _3_ –CH _3_ NH _3_ PbI _3_ tandem solar cells. Energy Environ. Sci..

[cit58] Gil-Escrig L. (2022). *et al.*, Perovskite/Perovskite Tandem Solar Cells in the Substrate Configuration with Potential for Bifacial Operation. ACS Mater. Lett..

[cit59] Roß M. (2020). *et al.*, Co-Evaporated p-i-n Perovskite Solar Cells Beyond 20% Efficiency: Impact of Substrate Temperature and Hole-Transport Layer. ACS Appl. Mater. Interfaces.

[cit60] Chiang Y.-H., Anaya M., Stranks S. D. (2020). Multisource Vacuum Deposition of Methylammonium-Free Perovskite Solar Cells. ACS Energy Lett..

[cit61] Borchert J. (2017). *et al.*, Large-Area, Highly Uniform Evaporated Formamidinium Lead Triiodide Thin Films for Solar Cells. ACS Energy Lett..

[cit62] Hörantner M. T. (2017). *et al.*, The Potential of Multijunction Perovskite Solar Cells. ACS Energy Lett..

[cit63] Kroll M. (2022). *et al.*, Insights into the evaporation behaviour of FAI: material degradation and consequences for perovskite solar cells. Sustainable Energy Fuels.

[cit64] Borchert J. (2019). *et al.*, Impurity Tracking Enables Enhanced Control and Reproducibility of Hybrid Perovskite Vapor Deposition. ACS Appl. Mater. Interfaces.

[cit65] de Wolf S. (2014). *et al.*, Organometallic Halide Perovskites: Sharp Optical Absorption Edge and. J. Phys. Chem. C.

[cit66] Cho C. (2022). *et al.*, Efficient vertical charge transport in polycrystalline halide perovskites revealed by four-dimensional tracking of charge carriers. Nat. Mater..

[cit67] Bi D. (2016). *et al.*, Efficient luminescent solar cells based on tailored mixed-cation perovskites. Sci. Adv..

[cit68] Kim Y. C. (2016). *et al.*, Beneficial Effects of PbI2 Incorporated in Organo-Lead Halide Perovskite Solar Cells. Adv. Energy Mater..

[cit69] Jacobsson T. J. (2016). *et al.*, Unreacted PbI _2_ as a Double-Edged Sword for Enhancing the Performance of Perovskite Solar Cells. J. Am. Chem. Soc..

[cit70] Doherty T. A. S. (2021). *et al.*, Stabilized tilted-octahedra halide perovskites inhibit local formation of performance-limiting phases. Science.

[cit71] Kosar S. (2021). *et al.*, Unraveling the varied nature and roles of defects in hybrid halide perovskites with time-resolved photoemission electron microscopy. Energy Environ. Sci..

[cit72] Merdasa A. (2019). *et al.*, Impact of Excess Lead Iodide on the Recombination Kinetics in Metal Halide Perovskites. ACS Energy Lett..

[cit73] Fu F. (2019). *et al.*, I 2 vapor-induced degradation of formamidinium lead iodide based perovskite solar cells under heat–light soaking conditions. Energy Environ. Sci..

[cit74] Holovský J. (2019). *et al.*, Lead Halide Residue as a Source of Light-Induced Reversible Defects in Hybrid Perovskite Layers and Solar Cells. ACS Energy Lett..

[cit75] Tumen-Ulzii G. (2020). *et al.*, Detrimental Effect of Unreacted PbI2 on the Long-Term Stability of Perovskite Solar Cells. Adv. Mater..

[cit76] Roose B., Dey K., Chiang Y. H., Friend R. H., Stranks S. D. (2020). Critical Assessment of the Use of Excess Lead Iodide in Lead Halide Perovskite Solar Cells. J. Phys. Chem. Lett..

[cit77] Chiang Y.-H., Anaya M., Stranks S. D. (2020). Multisource Vacuum Deposition of Methylammonium-Free Perovskite Solar Cells. ACS Energy Lett..

[cit78] Lohmann K. B. (2020). *et al.*, Control over Crystal Size in Vapor Deposited Metal-Halide Perovskite Films. ACS Energy Lett..

[cit79] Zheng Z. (2022). *et al.*, Development of formamidinium lead iodide-based perovskite solar cells: efficiency and stability. Chem. Sci..

[cit80] Fitzsimmons M. (2025). *et al.*, Optimized graphene-oxide-based interconnecting layer in all-perovskite tandem solar cells, under revision. ACS Energy Lett..

[cit81] Yang Z. (2019). *et al.*, Enhancing electron diffusion length in narrow-bandgap perovskites for efficient monolithic perovskite tandem solar cells. Nat. Commun..

[cit82] Xiao K. (2020). *et al.*, All-perovskite tandem solar cells with 24.2% certified efficiency and area over 1 cm2 using surface-anchoring zwitterionic antioxidant. Nat. Energy.

[cit83] Li C. (2020). *et al.*, Low-bandgap mixed tin–lead iodide perovskites with reduced methylammonium for simultaneous enhancement of solar cell efficiency and stability. Nat. Energy.

[cit84] Liu H. (2021). *et al.*, Modulated Crystallization and Reduced VOC Deficit of Mixed Lead–Tin Perovskite Solar Cells with Antioxidant Caffeic Acid. ACS Energy Lett..

[cit85] Kapil G. (2021). *et al.*, Tin-Lead Perovskite Fabricated *via* Ethylenediamine Interlayer Guides to the Solar Cell Efficiency of 21.74%. Adv. Energy Mater..

[cit86] Lin R. (2022). *et al.*, All-perovskite tandem solar cells with improved grain surface passivation. Nature.

[cit87] Mercaldo L. V. (2022). *et al.*, Procedure Based on External Quantum Efficiency for Reliable Characterization of Perovskite Solar Cells. Energy Technol..

[cit88] Saliba M., Unger E., Etgar L., Luo J., Jacobsson T. J. (2023). A systematic discrepancy between the short circuit current and the integrated quantum efficiency in perovskite solar cells. Nat. Commun..

[cit89] Wang J., Bi L., Fu Q., Jen A. K.-Y. (2024). Methods for Passivating Defects of Perovskite for Inverted Perovskite Solar Cells and Modules. Adv. Energy Mater..

[cit90] Xia J. (2023). *et al.*, Surface Passivation Toward Efficient and Stable Perovskite Solar Cells. Energy Environ. Mater..

[cit91] Zhang Z. (2023). *et al.*, Rationalization of passivation strategies toward high-performance perovskite solar cells. Chem. Soc. Rev..

[cit92] Kim J., Ho-Baillie A., Huang S. (2019). Review of Novel Passivation Techniques for Efficient and Stable Perovskite Solar Cells. Sol. RRL.

[cit93] Boccard M., Ballif C. (2020). Influence of the Subcell Properties on the Fill Factor of Two-Terminal Perovskite–Silicon Tandem Solar Cells. ACS Energy Lett..

[cit94] Saliba M., Stolterfoht M., Wolff C. M., Neher D., Abate A. (2018). Measuring Aging Stability of Perovskite Solar Cells. Joule.

[cit95] Morales-Masis M., De Wolf S., Woods-Robinson R., Ager J. W., Ballif C. (2017). Transparent Electrodes for Efficient Optoelectronics. Adv. Electron. Mater..

[cit96] Transparent Conductive Zinc Oxide: Basics and Applications in Thin Film Solar Cells, ed. K. Ellmer, A. Klein and B. Rech, Springer, Berlin, Heidelberg, 2008, vol. 104

[cit97] Wang E. Y. , Yu F. T. S., Sims V. L., Brandhorst E. W. and Broder J. D., Optimum Design of Anti-reflection Coating for Silicon Solar Cells, 1973, pp. 168–171

[cit98] Yang T. C.-J., Fiala P., Jeangros Q., Ballif C. (2018). High-Bandgap Perovskite Materials for Multijunction Solar Cells. Joule.

[cit99] Igual-Muñoz A. M., Ávila J., Boix P. P., Bolink H. J. (2020). FAPb0.5Sn0.5I3: A Narrow Bandgap Perovskite Synthesized through Evaporation Methods for Solar Cell Applications. Sol. RRL.

[cit100] Pitaro M. (2023). *et al.*, A carbazole-based self-assembled monolayer as the hole transport layer for efficient and stable Cs0.25FA0.75Sn0.5Pb0.5I3 solar cells. J. Mater. Chem. A.

[cit101] Rühle S. (2016). Tabulated values of the Shockley–Queisser limit for single junction solar cells. Sol. Energy.

