# Supplementary material for: Incorporating thermal co-evaporation in current-matched all-perovskite triple-junction solar cells
Source: EES Solar. 2025 Jan 21;1(1):41–55. doi: 10.1039/d4el00012a (PMC11755821; doi:10.1039/d4el00012a)
Supplement: EL-001-D4EL00012A-s001 [file EL-001-D4EL00012A-s001.pdf]

Supplementary Information (SI) for EES Solar.
This journal is © The Royal Society of Chemistry 2025

Supplementary Information for:

Incorporating Thermal Co-evaporation in Current-Matched All-Perovskite Triple-Junction Solar Cells

Authors: Terry Chien-Jen Yang[1,2,#], Taeheon Kang[1,#], Melissa Fitzsimmons[1], Guadalupe Vega[3], Yang Lu[1,2], Leo Rosado[4], Alberto Jiménez-Solano[5,6], Linfeng Pan[1], Szymon J. Zelewski[1,2,7], Jordi Ferrer Orri[1,2,8], Yu-Hsien Chiang[1,2], Dengyang Guo[1,2], Zher Ying Ooi[1], Yutong Han[1], Weidong Xu[1], Bart Roose[1,2], Caterina Ducati[8], Sol Carretero Palacios[4], Miguel Anaya[1,3], Samuel D. Stranks[1,2,*]

1. Department of Chemical Engineering and Biotechnology, University of Cambridge, Cambridge CB3 0AS, UK.
2. Department of Physics, Cavendish Laboratory, University of Cambridge, Cambridge CB3 0HE, UK.
3. Instituto de Ciencia de Materiales de Sevilla, Universidad de Sevilla−CSIC, Calle Américo Vespucio 49, Sevilla 41012, Spain.
4. Instituto de Ciencia de Materiales de Madrid, ICMM-CSIC, 28049 Madrid, Spain.
5. Max Planck Institute for Solid State Research, Heisenbergstrasse 1, 70569 Stuttgart, Germany.
6. Departamento de Física, Universidad de Córdoba, Edificio Einstein (C2), Campus de Rabanales, 14071 Córdoba, Spain.
7. Department of Experimental Physics, Faculty of Fundamental Problems of Technology, Wrocław University of Science and Technology, 50-370 Wrocław, Poland.
8. Department of Materials Science and Metallurgy, University of Cambridge, Cambridge, CB3 0FS, UK.

[#] These authors contributed equally to the work

[*] e-mail: sds65@cam.ac.uk

Supplementary Note 1:

1. Basic theory for monolithic (series-connected) multijunction solar cells

In terms of the basic theory, for any monolithic (series-connected) multijunction (MJ) solar cell, in the ideal case, the V_{OC} of the entire device, $V_{OC\text{multi}}$ can be approximated by summing the individual V_{OC} values of each subcell, V_{OCi}, as shown in equation (1). Note, this should not be confused with simply adding the V_{OC} values of the single-junction solar cell equivalents because in a MJ solar cell the subcells below the top subcell will receive less light (more details in the next section).

$$V_{OCmulti} = \sum_{i=1}^{n} V_{OCi} \tag{1}$$

The final J_{SC} of a monolithic MJ solar cell is determined by the subcell with the lowest current, as shown in equation (2). This can typically be found by the standard *J-V* measurement of the device. However, for a monolithic MJ solar cell it is not possible to obtain the J_{SC} of each individual subcell directly from the *J-V* measurement because there are only 2-terminals (this is only possible in mechanically stacked MJ solar cells where separate contacts for each subcell are available). In order to find the individual J_{SC} values of each subcell in a monolithic MJ solar cell, external quantum efficiency measurements (EQE) are needed.

$$J_{SCmulti} = \min_{i=1} J_{SCi} \tag{2}$$

Equation (3) shows the EQE_i of a subcell in a MJ solar cell, which is the ratio between number of electron-hole pairs generated versus the incident number of photons and is simply a ratio between the J_{SC} of a subcell, $J_{SCi}(\lambda)$ divided by the photon flux, ϕ_i (λ). Thus, in terms of finding the J_{SC} of each individual subcell, J_{SCi}, it is simply a matter of integrating the EQE curves with respect to the wavelength, λ.

$$EQE_i(\lambda) = \frac{J_{SCi}(\lambda)}{\phi_i(\lambda)}\text{, where} \quad J_{SCi} = \int_{\lambda_1}^{\lambda_2} q\phi_i(\lambda) EQE_i(\lambda) d\lambda \tag{3}$$

The fundamental understanding of the physics theory of MJ solar cells is important in order to optimise its performance. In general, there are a number of ways to optimise the performance in a MJ solar cell, (1) finding the optimum bandgap absorbers in terms of thickness versus absorption and extraction of the charges, (2) finding alternative materials that reduce the parasitic absorption whilst still maintaining functionality, (3) reducing the bulk and surface recombination of the layers in the stack, especially at the recombination/tunnel-junction interconnections (4) optimise the light trapping through better photon management. This understanding aids us in the design of an effective monolithic MJ solar cell. Other than improving the V_{OC} of each of the individual subcells, it is important to make sure that the J_{SC} of each of the individual subcells match and are as high as possible (this is firstly dictated by the bandgap of the bottom subcell). For example, our single-junction Pb-Sn solar cell (around 800 nm in thickness) has a J_{SC} of just under 30 mA/cm^2. This means that if we make a monolithic tandem (two-junction) with a high-bandgap perovskite absorber as the top subcell, the maximum current-matched device would have a J_{SC} of 15 mA/cm^2, similarly for a triple-junction that value would be 10 mA/cm^2. In reality however, there would be parasitic optical or resistive losses in many of the functional layers above the bottom Pb-Sn subcell such as electron/hole transport, interconnection/recombination or tunnel-junction, and/or even passivation layers.

2. Discussion on V_{OC} additions in multijunction solar cells.

For a solar cell, the open-circuit voltage, V_{OC} is the maximum voltage available to it, which occurs when the current is zero. The V_{OC} corresponds to the amount of forward bias on the solar cell due to the bias of the solar cell junction with the light-generated current. Supplementary Fig. 1 shows the IV curve with the V_{OC}.

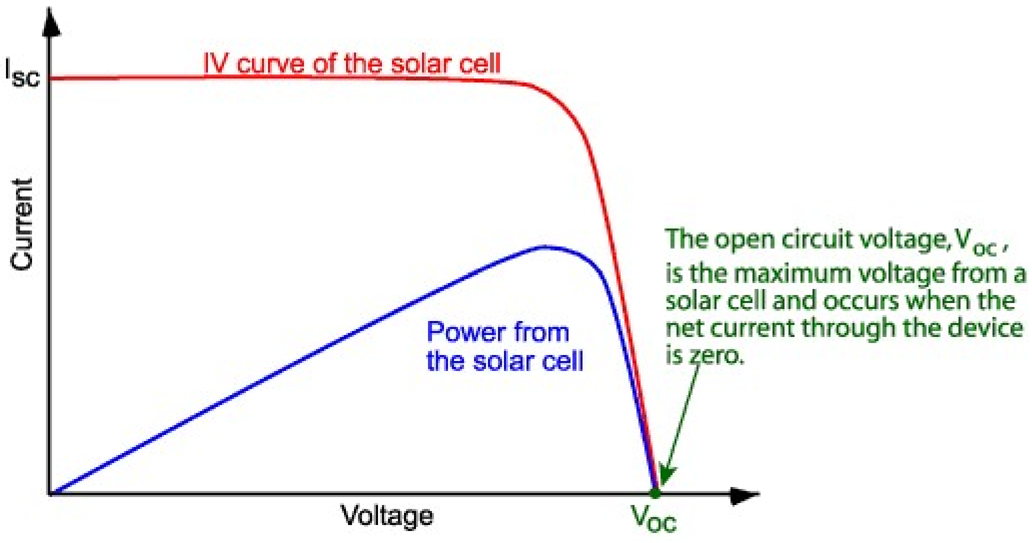

Supplementary Fig. 1 | *I-V* and *P-V* curves of a solar cell showing the open-circuit voltage, V_{OC}[1].

The equation for V_{OC} can be found by setting the net current equal to zero in the solar cell equation (4) below:

$$V_{OC} = \frac{nkT}{q} ln\left(\frac{I_L}{I_0} + 1\right) \quad (4)$$

where *n* is the ideality factor, *k* is the Boltzmann constant, *T* is the absolute temperature in Kelvin, *q* is the elementary charge, I_L is the light generated current, and I_0 is the dark saturation current.

Now, at 300 K, the prefix term *kT/q* = 25.85 mV (excluding the ideality factor, *n*), is known as the "thermal voltage“. From the thermal voltage, it is known that for every order of magnitude increase in I_L and given the ln term in the second half of equation (4), the V_{OC} of the solar cell should increase by approximately 60 mV.

Referring to the triple-junctions in the main text, subsequent subcells will receive less light versus their single-junction equivalents under the same 1-sun illumination since light has to pass through the higher subcell(s) above them first which will already have absorbed a portion of the incoming light. For example, assuming in an ideal case of a perfect monolithic perovskite-perovskite-

perovskite (PPP) triple-junction solar cell (with no parasitic losses), where the bottom Pb-Sn single-junction device yields a short-circuit current of approximately 30 mA/cm^2 and the EQE curves for each subcell resemble a “top hat” profile with each of the top, middle, and bottom subcells receiving an equal 10 mA/cm^2. In the ideal case (without any light trapping, photon recycling, and other effects), if the subcells had the same thickness as their single-junction equivalents, then:

1. the top cell single-junction equivalent should have the same V_{OC} as its subcell in the triple-junction.
2. The middle cell single-junction equivalent however in the triple-junction would have lost about 1/3 of its light to the top subcell first, meaning that its V_{OC} in the triple-junction as the middle subcell would be approximately 11 mV lower.
3. The bottom cell single-junction equivalent in the triple-junction would have lost 2/3 of its light to the top and middle subcells, meaning that its V_{OC} in the triple-junction as the bottom subcell would be approximately 29 mV lower.

Note, this is simply a rough calculation based the ideal case where there is no light scattering or recycling effects and full ideal absorption profile case. In reality, there are many factors involved that would govern the value of the final V_{OC} in a full device, especially one as complicated as a PPP triple-junction solar cell.

Supplementary Note 2

1. Literature Review Comparison with Existing All-Perovskite PPP Triple-Junction Solar Cells

We conducted a literature review of existing monolithic (series-connected) triple-junction PPP solar cells published since Aug 2018. So far there have been a total of 4 publications in PPP monolithic triple-junction solar cells. What is interesting is the examination of the bandgap values (in some cases authors used absorption edge values) of the top, middle, and bottom subcells in the stack. In terms of transfer matrix modelling (TMM), Hörantner et al.[2] (2017) through heatmaps showed that for PPP triple-junction solar cells, the optimum efficiency (33.0%) occurs when the top and middle subcells are 2.05 and 1.59 eV (bottom subcell is fixed at 1.22 eV). The heatmaps suggest that from the peak efficiency points there is vast room for bandgap variation without severe loss in efficiency achievable. The model was done with the common perovskite absorbers and interlayer materials used at the time (for example, hole/electron transport, transparent conductive oxide, and others), although improvements in device design and new materials have been made since 2017. One interesting outlook is that more recently, it was shown by Bowman et al.[3] that luminescence coupling or photon recycling, often associated with high external radiative materials like perovskites[4–6], has added benefits such as increased flexibility in subcell thicknesses and tolerance to different spectral conditions. Furthermore, this means that the wider bandgap of the top subcells can in fact be lower meaning that unstable bandgap compositions prone to photoinduced halide phase segregation (PIHPS)[7–18] can be avoided.

Supplementary Fig. 2a shows a graph of all the bandgaps used for the subcells in the different monolithic triple-junction PPP solar cells published since 2018. It is quite interesting to note that the choice of the top cell perovskite bandgap across all devices have the largest range (2.00 to 1.73 eV, highlighted in blue), whereas the middle (1.60 to 1.50 eV, highlighted in green) and bottom (1.34 to 1.22 eV, highlighted in red) have smaller ranges. The recent work from Wang et al.[19] used inorganic Cs-Rb top subcells to mitigate the effect of PIHPS, allowing V_{OC} values of over 1.3 V whilst

maintaining high J_{SC} for the entire device. From Supplementary Fig. 2b, the integrated subcell EQE, that is the EQE-J_{SC} values for each subcell from each reference is shown. What is interesting to note is the spread between the three values, where the wider the spread, the more loss and wastage in current in the series-connected monolithically stacked devices due to the current-mismatch. This is typical of earlier PPP devices, it seems that there is clear improvement in terms of the spread since the first device was demonstrated by McMeekin et al. in 2019[20], as perovskite processing and deposition methods are improving as well as the research effort and resources.

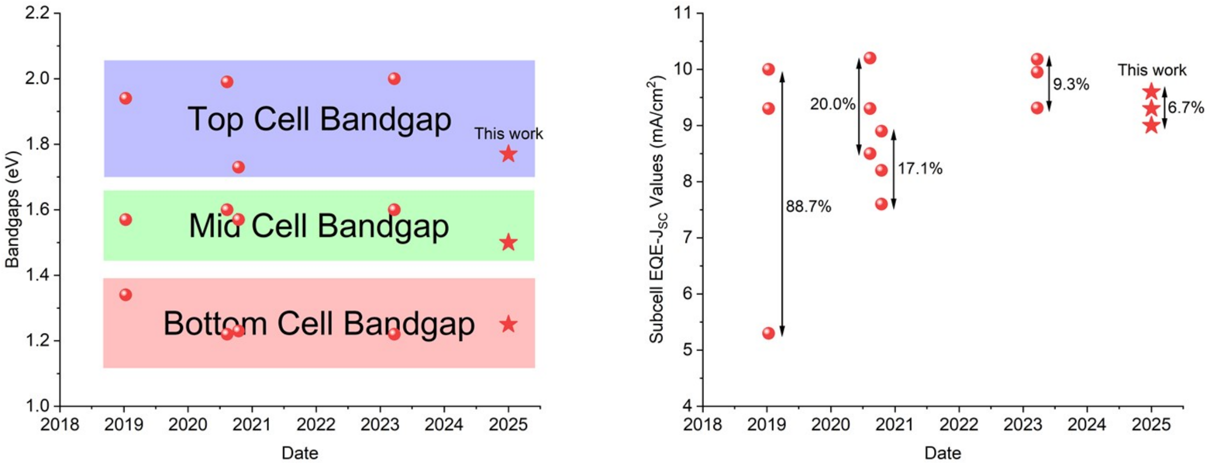

Supplementary Fig. 2 | Literature review of the bandgap and EQE-J_{SC} values of existing perovskite-perovskite-perovskite monolithic (series connected) triple-junction solar cells by date. a. Bandgaps, and **b.** EQE-J_{SC} of the top, middle, and bottom subcells plotted by date of publication.

Supplementary Fig. 3a-d shows the V_{OC}, J_{SC}, FF, and PCE values respectively of all the published monolithic PPP triple-junction solar cells since 2018. The V_{OC} in literature are all above 2.7 V, whereas our champion device only had a V_{OC} of 2.38 V. The reasons for which have been outlined in the main text. In terms of J_{SC} however, our device was able to achieve the second highest value of 9.3 mA/cm^2, after the recent work by Wang et al.[19]. We attribute this to our excellent bandgap and thickness management that comes with the thermal co-evaporation process as well as the change from ALD-SnO_x/Au/PEDOT:PSS/PTAA to ALD-SnO_x/GO/MeO-2PACz (for the top-middle interconnect)

and ALD-SnO_x/Au/PEDOT:PSS to ALD-SnO_x/GO/2PACz (for the middle-bottom interconnect). The FF values from existing published triple-junction devices also showed a large range. Disregarding the data point at 2018, all the monolithic PPP triple-junction solar cells had a FF of 68% or more, which is promising to see. Of course, naturally MJ solar cells will allow for a higher FF given the larger summed V_{OC} which affects the *J-V* curve geometrically. However, another explanation is that for MJ solar cells, especially triple-junctions in some cases may have higher shunt resistances due to the application of less conductive layers such as the ALD-SnO. On the contrary, if any local regions have low shunt resistance, then this will affect the the triple-junction device far more than the single-junction counterparts. More in depth theory on FF dependence in tandem/multijunctions is discussed in the work by Boccard and Ballif[21]. Wang et al.[19] holds the efficiency record for monolithic PPP triple-junction solar cells. They achieved this through the optimisation of their top subcell consisting of an inorganic combination of Cs and Rb. Their $Rb_{0.15}Cs_{0.85}PbI_{1.75}Br_{1.25}$ single-junction devices were able to achieve V_{OC} >1.3 V and thus the entire triple-junction stack was able to achieve a V_{OC} > 3.2 V. In addition to careful bandgap and thickness management a high efficiency of 24.3% could be achieved. Of course, there is still much more room for improvement as the triple-junction solar cell efficiencies are still much lower than the record PP or PS tandem solar cells reported, even though their theoretical efficiencies are higher.

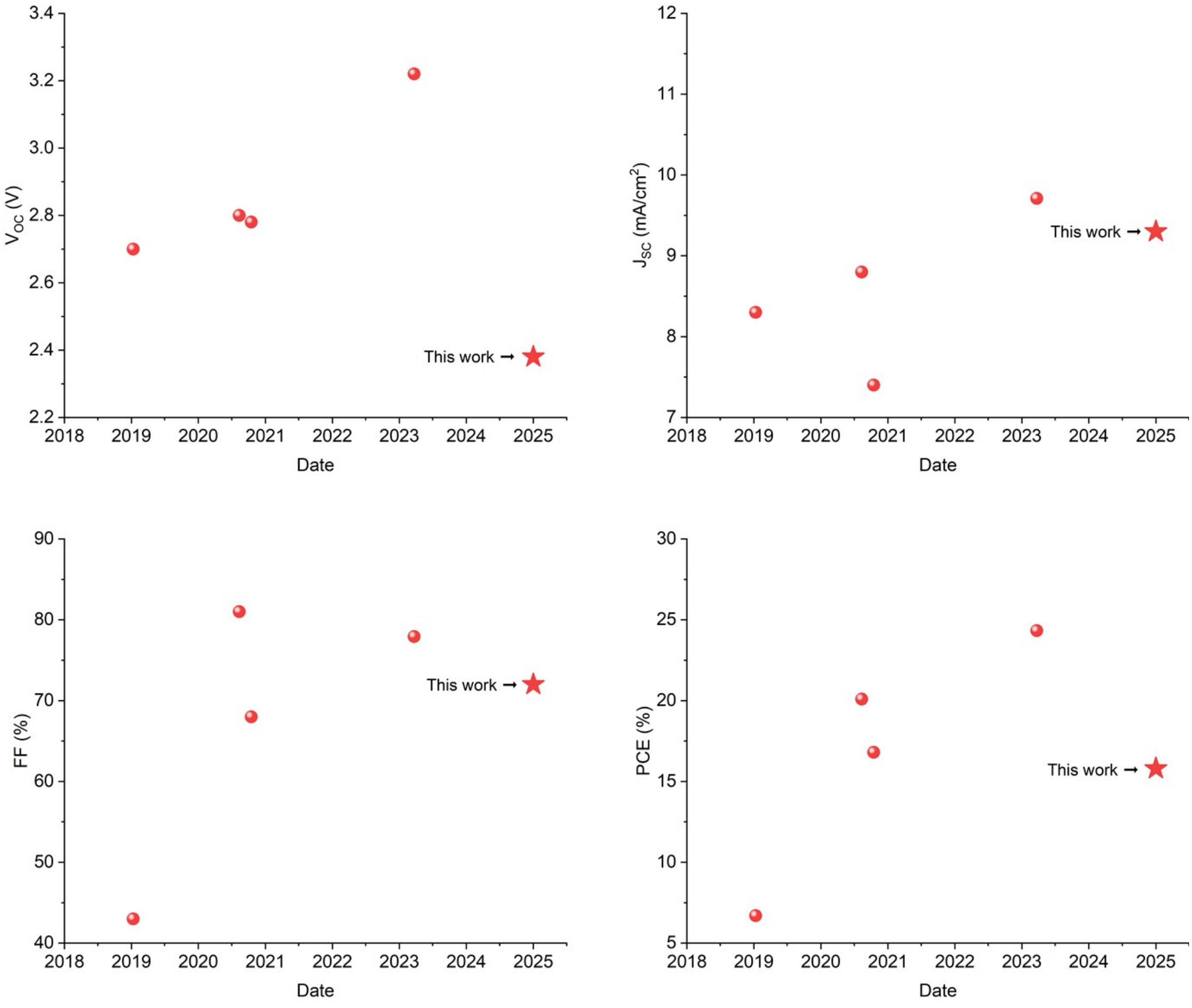

Supplementary Fig. 3 | Literature review of the *J-V* parameters of existing PPP triple-junction solar cells by date. a. V_{OC}, b. J_{SC}, c. FF, and d. PCE.

In terms of the active area, in our case we fabricated devices with a circular copper contact area of 0.146 cm^2 with a diameter of 4.32 mm and a measurement active area of 0.118 cm^2 using a circular metal mask with diameter of 3.88 mm. Even though our champion efficiency here is not the highest reported at a modest 15.8%, to our knowledge our measurement active area of 0.118 cm^2 is the largest active area reported to date for all PPP triple-junction solar cells, the data of which is plotted in Supplementary Fig. 4.

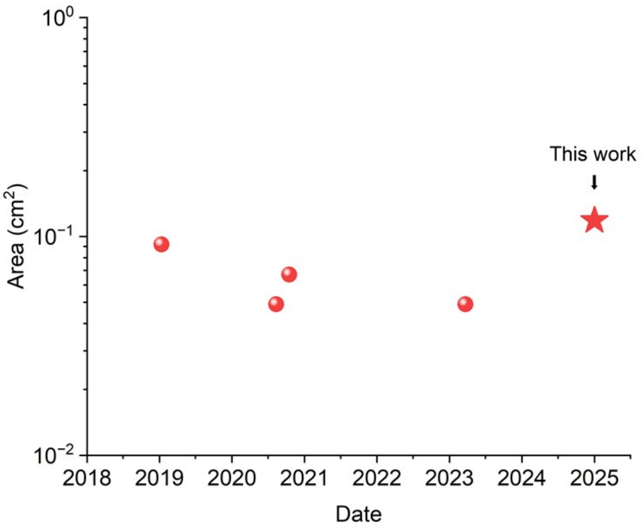

Supplementary Fig. 4 | Literature review of the measurement area of perovskite-based triple-junction solar cells.

Supplementary Table 1 | Literature Review of PPP monolithic triple-junction solar cells.

High Bandgap	Interface	Middle Bandgap	Interface	Bottom Bandgap	V_{OC} (V)	J_{SC} (mA/cm²)	FF (%)	PCE (%)	Area (cm²)	Date Accepted
$FA_{0.83}Cs_{0.17}Pb(Br_{0.7}I_{0.3})_3$ 1.94	PEDOT:PSS/ITO NPs	$MAPbI_3$ (1.57 eV) 1.57	PEDOT:PSS/ITO NPs	$MAPb_{0.75}Sn_{0.25}I_3$ 1.34	2.70	8.3	43	6.7	0.092	11/01/2019
$Cs_{0.2}FA_{0.8}PbI_{0.9}Br_{2.1}$ 1.99	ALD-SnO_2/Au	$Cs_{0.05}FA_{0.95}PbI_{2.55}Br_{0.45}$ 1.6	ALD-SnO_2/Au	$MA_{0.3}FA_{0.7}Pb_{0.5}Sn_{0.5}I_3$ 1.22	2.80	8.8	81	20.1	0.049	11/08/2020
$Cs_{0.1}(FA_{0.66}MA_{0.34})_{0.9}PbI_2Br$ 1.73	ALD-SnO_2/Au	$FA_{0.66}MA_{0.34}PbI_{2.85}Br_{0.15}$ 1.57	ALD-SnO_2/Au	$FA_{0.66}MA_{0.34}Pb_{0.5}Sn_{0.5}I_3$ 1.23	2.78	7.4	68	14	0.067	16/10/2020
$Rb_{0.15}Cs_{0.85}PbI_{1.75}Br_{1.25}$ 2	ALD-SnO_x/ITO	$Cs_{0.05}FA_{0.9}MA_{0.05}Pb(I_{0.9}Br_{0.1})_3$ 1.6	ALD-SnO_2/Au	$Cs_{0.05}FA_{0.7}MA_{0.25}Pb_{0.5}Sn_{0.5}I_3$ 1.22	3.22	9.71	77.93	24.33	0.049	23/03/2023
$Cs_{0.3}FA_{0.7}Pb(I_{0.56}Br_{0.44})_3$ 1.80	ALD-SnO_2/GO	$FAPbI_3$ 1.53	ALD-SnO_2/GO	$Cs_{0.25}FA_{0.75}Pb_{0.5}Sn_{0.5}I_3$ 1.25	2.38	9.3	72	15.8	0.118	1/01/2025

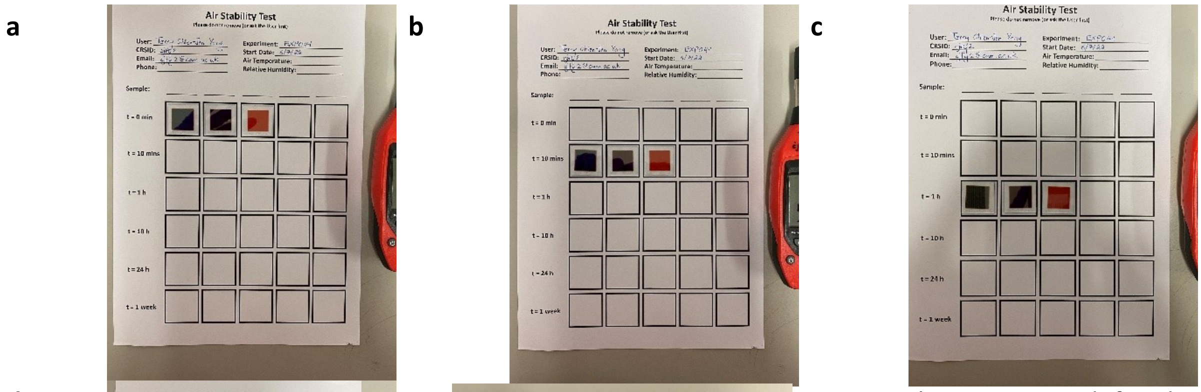

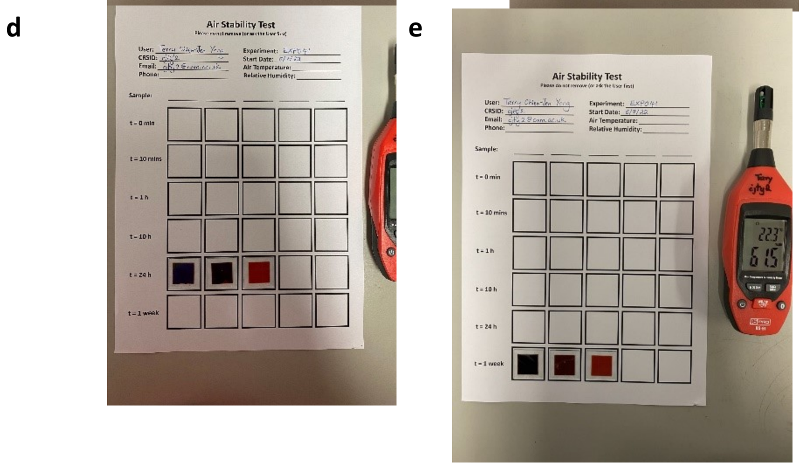

Sample variation is defined by the FAI rate from (left to right): 0.9, 1.2, 1.5 Å/s. The PbI_2 rate was fixed at 0.6 Å/s.

Supplementary Fig. 5. | Photographs of the thermally co-evaporated $FAPbI_3$ films on glass/ITO/MeO-2PACz over time. a. initial t = 0 h, **b.** t = 10 mins, **c.** 1 hour, **d.** 24 hours, **e**. 1 week in air with the humidity and temperature shown by the hygrometer on the right.

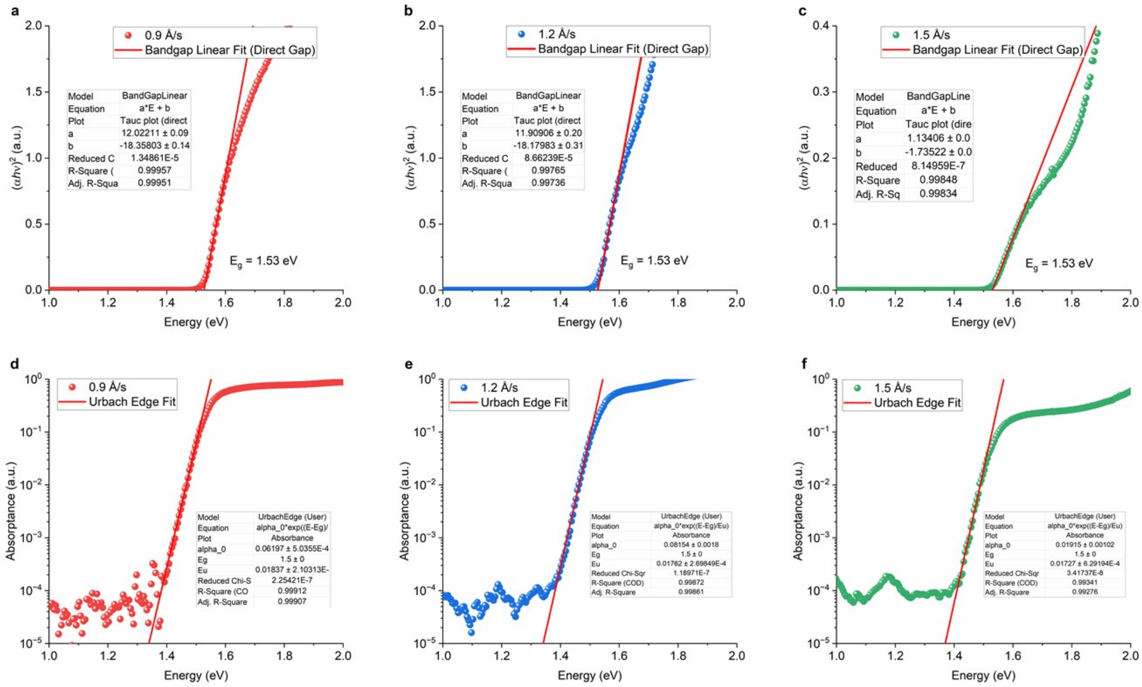

Supplementary Fig. 6. | Photothermal deflection spectroscopy data and analysis. Tauc plot and Urbach edge fits for the **a, d,** 0.9 Å/s, **b,e,** 1.2 Å/s, and **c,f,** 1.5 Å/s FAI rates respectively. The PbI_2 rate was fixed at 0.6 Å/s.

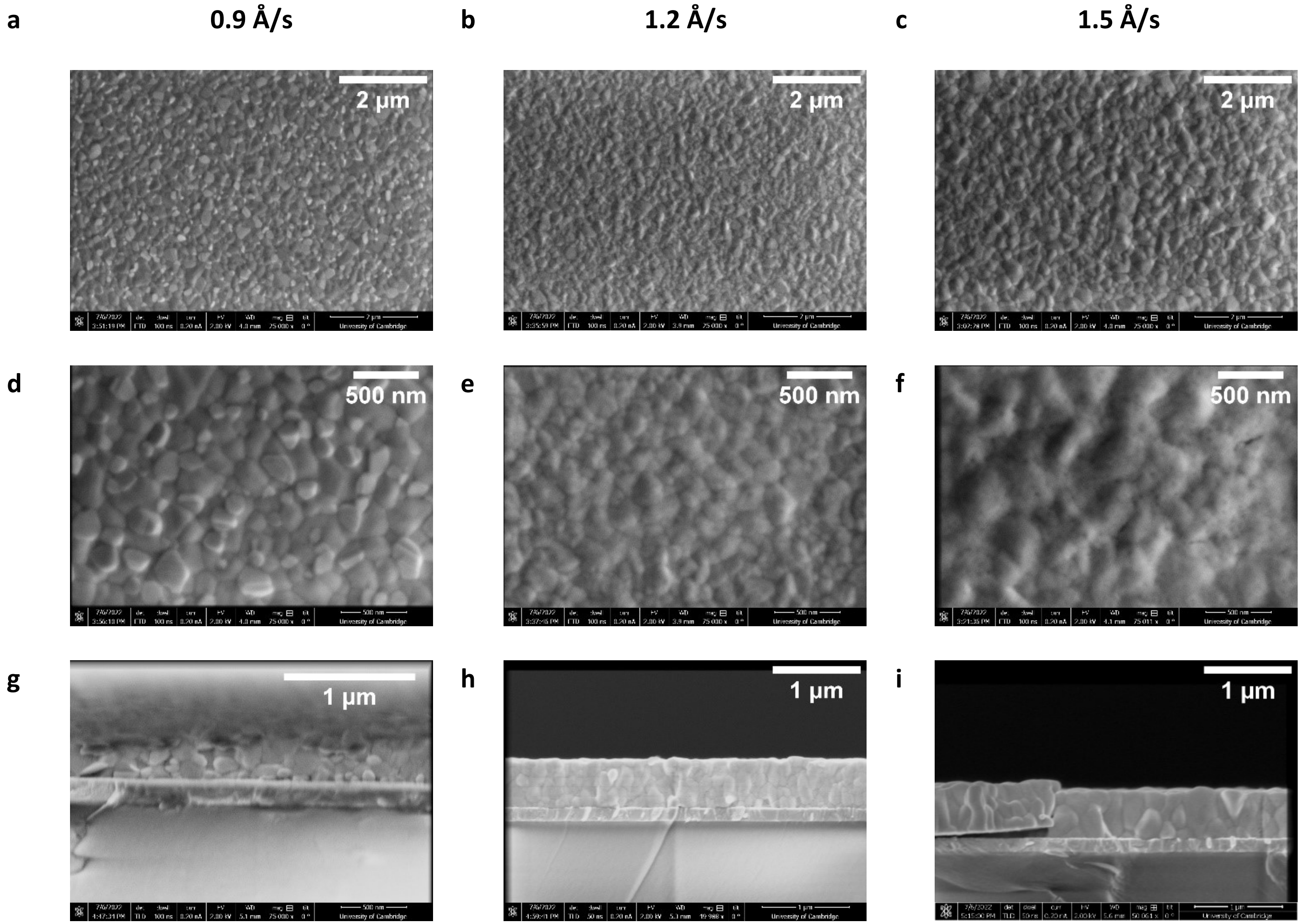

Supplementary Fig. 7 | Scanning electron microscopy images of the thermally co-evaporated $FAPbI_3$ samples on glass/ITO/MeO-2PACz. a, b, c top view (field of view (FOV): 8.5 µm), **d, e, f** top view (FOV: 2.8 µm)**, g, h, i,** cross-sectional view for the 0.9 (FOV: 2.8 µm), 1.2 (FOV: 4.1 µm), and 1.5 (FOV: 4.1 µm) FAI rate samples (where the PbI_2 rate was fixed at 0.6 Å/s).

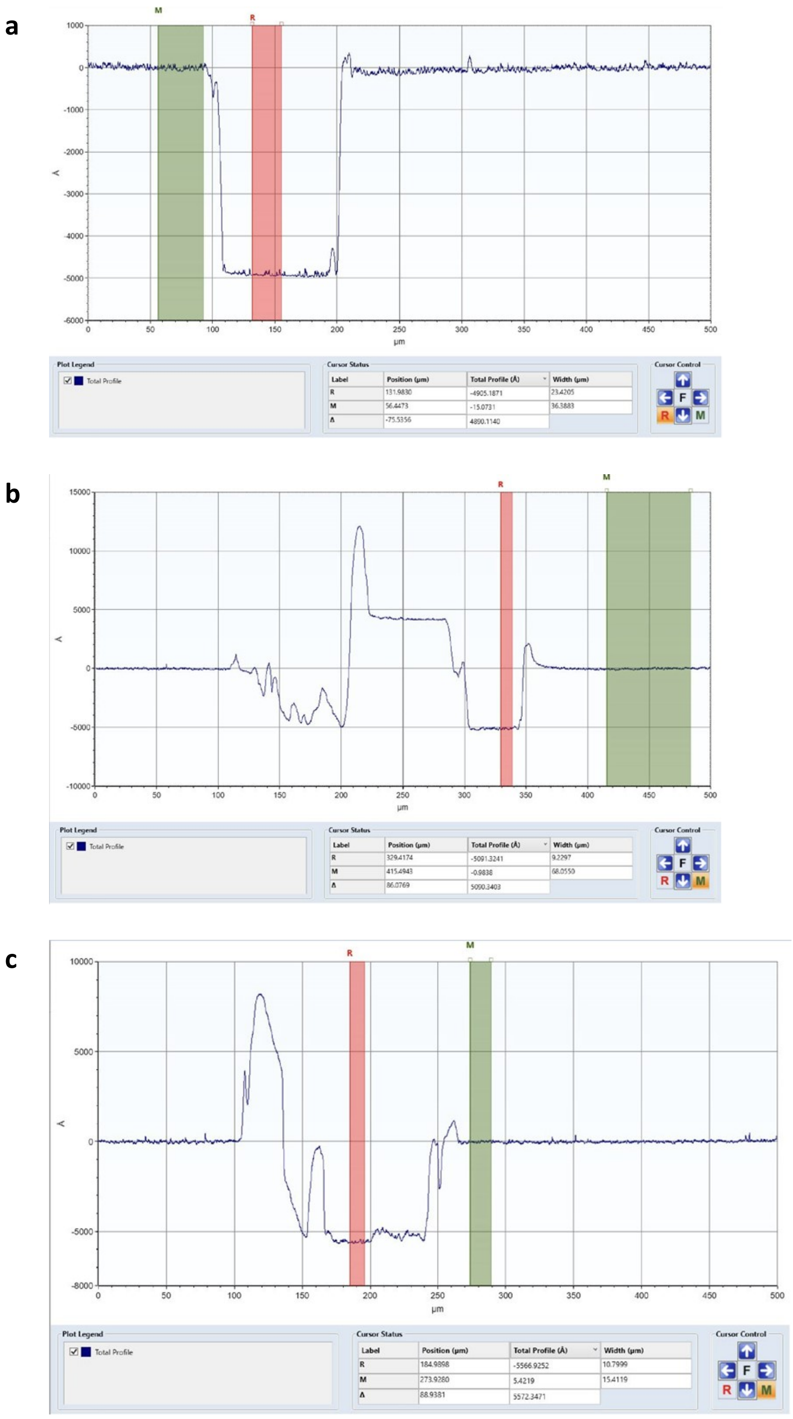

Supplementary Fig. 8 | Thickness measurement of thermally co-evaporated $FAPbI_3$ films on glass. Dektak profilometry of the $FAPbI_3$ samples on glass for various FAI rates where PbI_2 is fixed at 0.6 Å/s **a.** 0.9 Å/s**, b.** 1.2Å/s**,** and **c.** 1.5 Å/s**.** The average across six measurements taken for each sample 0.9 Å/s**,** 1.2Å/s**,** and 1.5 Å/s was 490, 512, and 552 nm respectively.

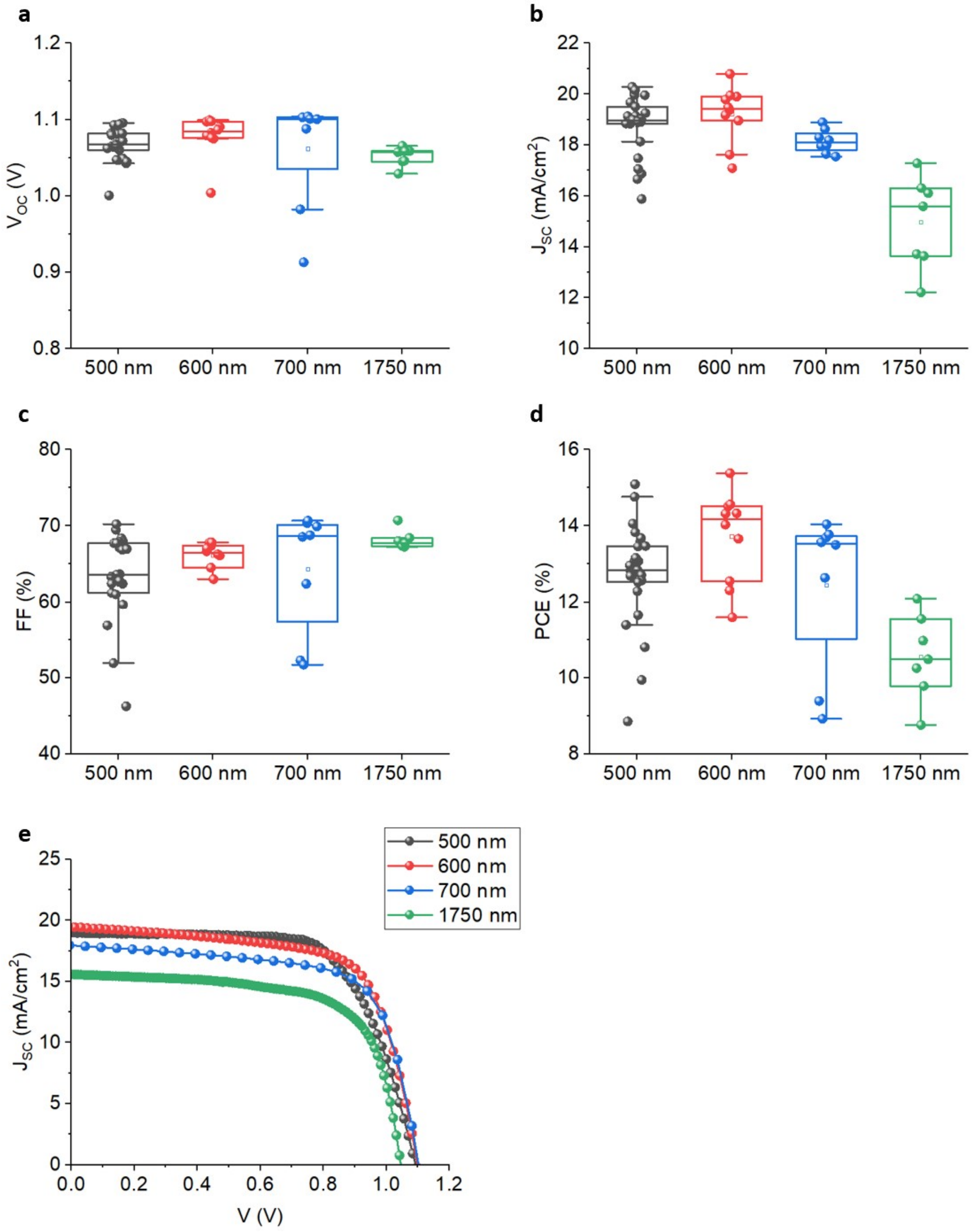

Supplementary Fig. 9 | PV parameters with thickness variation for the FAI = 0.9 A/s thermally co-evaporated $FAPbI_3$ PSCs. a. V_{OC}, **b.** J_{SC}, **c.** FF, **d.** PCE, and e representative J-V curves (reverse scan).

Supplementary Table 2 | Series and shunt resistances of thermally co-evaporated $FAPbI_3$ solar cells made with varying FAI evaporation rates (includes statistical analysis)

FAI evaporation rate (Å/s)	Series Resistance (Ω·cm^2)	Standard Deviation (Ω·cm^2)	Shunt Resistance (Ω·cm^2)	Standard Deviation (Ω·cm^2)
0.6	15.5	1.2	543.8	145.3
0.9	11.8	3.9	503.4	272.5
1.2	24.1	3.4	59.5	15.5
1.5	88.2	51.5	97.0	64.7

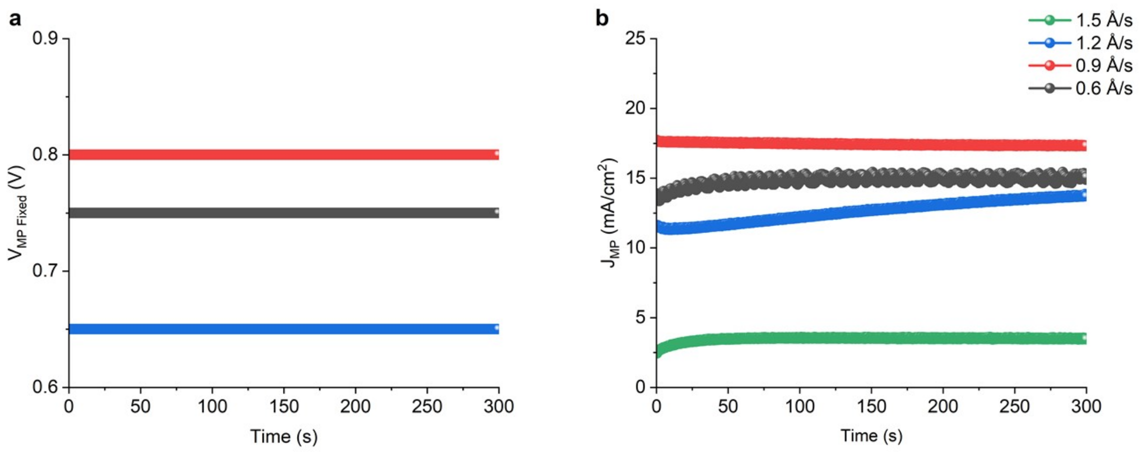

Supplementary Fig. 10 | *J-V* stability parameters. a. V_{MP} fixed at the initial optimum value, and **b.** J_{MP} over the course of 300 seconds.

Supplementary Note 3

Optical transfer matrix modelling and complex refractive index (dielectric function) data

Complex refractive index determination. We developed a model based on the transfer matrix method boosted by a genetic algorithm, that allowed us to extract the complex refractive index (*n + ik*) of the halide perovskite films of interest. We first extracted the complex refractive index of each composition by simultaneously fitting the ellipsometry data of three perovskite films of different thicknesses (using Forouhi-Bloomer[22] model) to ensure robustness. Then, we refined the calculated constants by fitting the experimental EQE of the perovskite solar cells in a single-junction configuration to account for variations in the response of the material when grown within a device architecture. The complex refractive index data for the three perovskite absorber films used in the monolithic PPP triple-junction solar cell can be found in Supplementary Fig. 11 on the next page. For the more general layers, such as the C60[23], ALD-SnO_x[24], ITO[25], Cu[26] and GO[27] complex refractive indices were taken from literature.

Optical design of the triple-junction device. Pareto statistics were utilised to minimise the difference between experimental and fitting curves by modifying the perovskite thicknesses while simultaneously maximising the current of each perovskite subcell. The calculated J_{SC} is determined by weighting the EQE by the AM 1.5 solar spectrum.

a
b
c

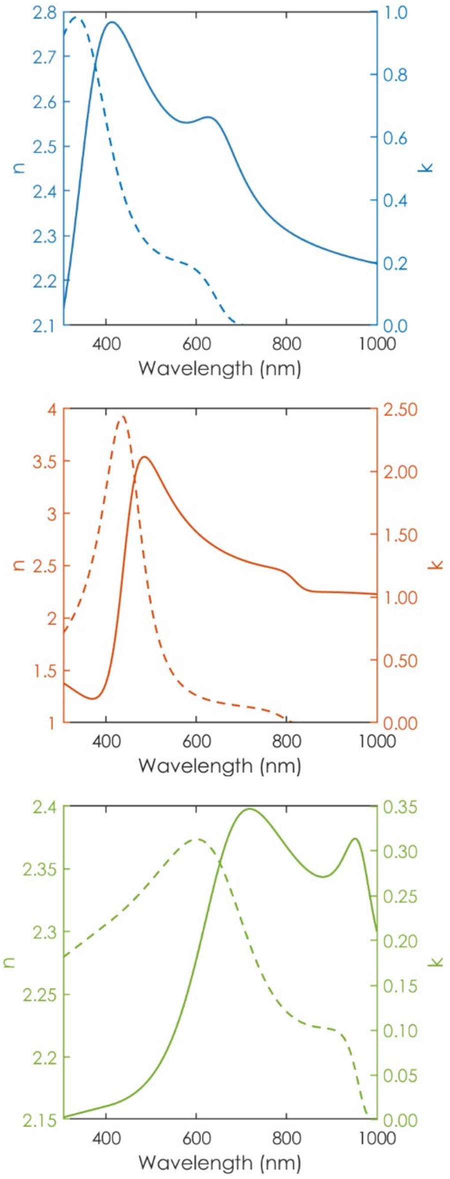

Supplementary Fig. 11 | Complex refractive index data. Real, *n* (solid line) and imaginary, *k* (dashed line) parts of the complex refractive index of **a.** $Cs_{0.3}FA_{0.7}Pb(I_{0.56}Br_{0.44})_3$ (top) **b.** $FAPbI_3$ (middle), and **c.** $Cs_{0.25}FA_{0.75}Pb_{0.5}Sn_{0.5}I_3$ (bottom) perovskites.

a

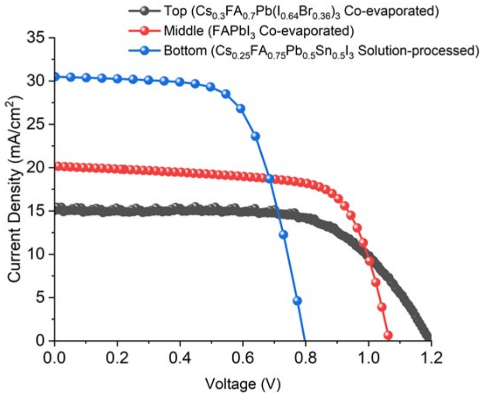

b

	V_{OC} (V)	J_{SC} (mA/cm^2)	FF (%)	PCE (%)
Top	1.20	15.05	64.63	11.63
Middle	1.07	20.16	70.22	15.09
Botto m	0.80	30.49	65.39	15.90

c

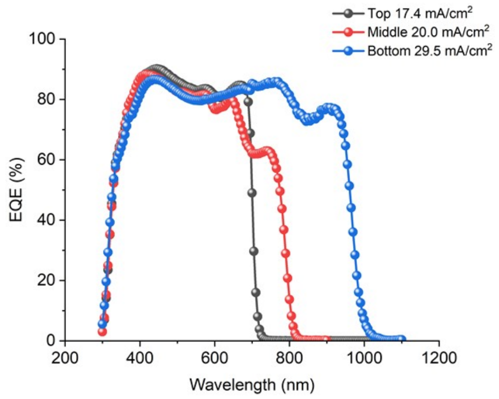

Supplementary Fig. 12 | Single-junction solar cell characteristics. **a.** *J-V* curves with the corresponding reverse *J-V* parameters shown in **b**, and **c.** EQE for the single-junction top, middle, and bottom solar cell equivalents, for use in the final monolithic PPP triple-junction solar cell.

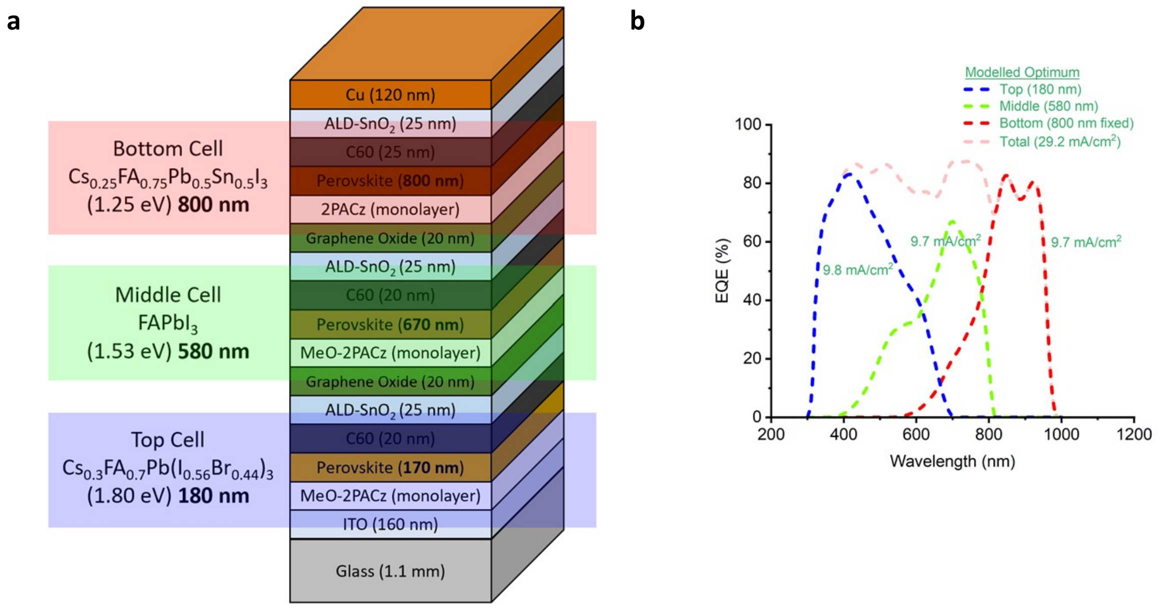

Supplementary Fig. 13 | Optical modelling of the experimentally demonstrated monolithic PPP triple-junction solar cell. a. Schematic diagram of the entire stack with thicknesses of the individual layers and the optical modelling optimisation ranges for the top and middle subcells. **b.** EQE for the maximum optically modelled point in the heatmap shown in Fig 2c of the main text.

a

b

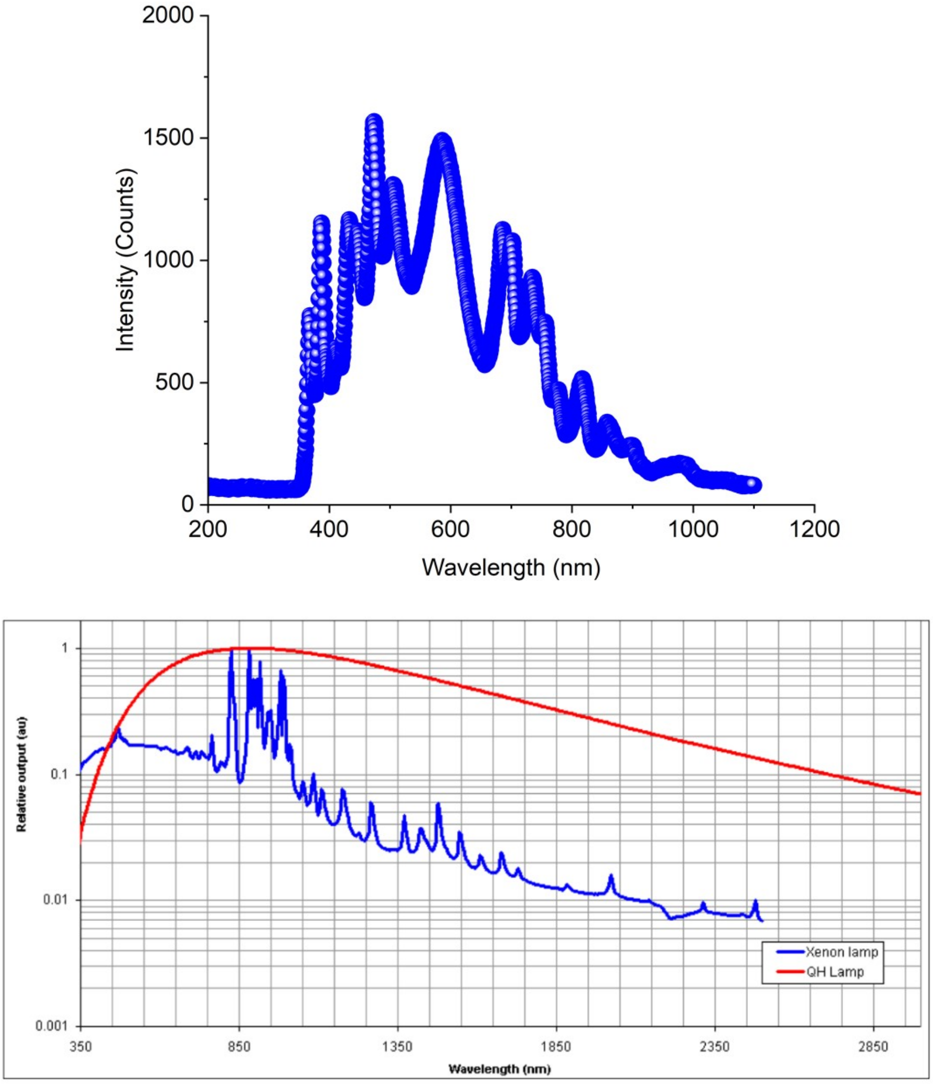

Supplementary Fig. 14 | *J-V* solar simulator and EQE lamp spectra. a. The measured AM1.5 spectrum from a class AAA LED solar simulator (G2V Optics) and **b.** EQE dual source lamp spectrum (xenon short arc and quartz halogen lamp). The EQE system (Bentham PVE300) produces a monochromatic probe source, 300-1800 nm.

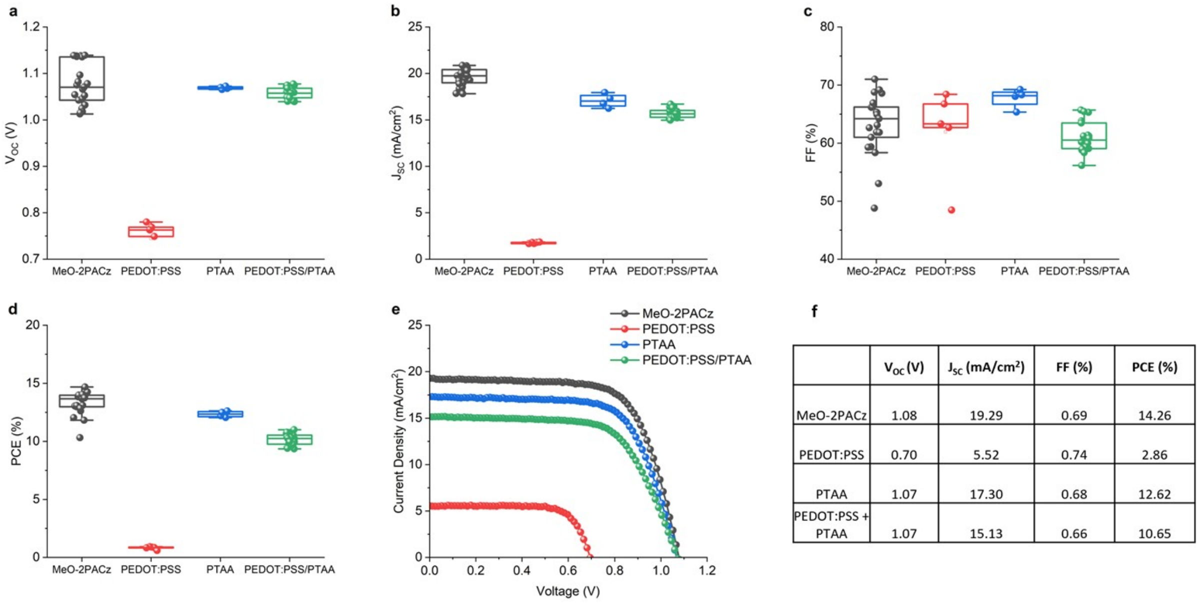

	V_{OC} (V)	J_{SC} (mA/cm^2)	FF (%)	PCE (%)
MeO-2PACz	1.08	19.29	0.69	14.26
PEDOT:PSS	0.70	5.52	0.74	2.86
PTAA	1.07	17.30	0.68	12.62
PEDOT:PSS + PTAA	1.07	15.13	0.66	10.65

Supplementary Fig. 15 | Testing of various hole transport layers for thermally co-evaporated $FAPbI_3$ solar cells. Four different HTLs or HTL combinations were tested MeO-2PACz, PEDOT:PSS, PTAA, and PEDOT:PSS/PTAA for thermally co-evaporated $FAPbI_3$ onto glass/ITO substrates, where **a.** V_{OC}, **b.** J_{SC}, **c.** FF**, d.** PCE**,** and **e.** representative *J-V* curves with corresponding reverse *J-V* parameters shown in **f.**

One of the first issues that needed to be tackled was the type of HTL available for the middle subcell, since the MeO-2PACz which we used for single-junction $FAPbI_3$ devices was not compatible with the ALD-SnO_2/Au interconnection between subcells in the MJ stack. MeO-2PACz or 2PACz cannot be deposited directly on top of the thin ohmic Au layer as it does not have any anchoring groups such as -OH in ITO[28]. Thus, we had to use an alternative form of HTL, we found that (poly[bis(4-phenyl)(2,4,6-trimethylphenyl)amine (PTAA) works reasonably well with the thermally co-evaporated $FAPbI_3$ process except for the higher parasitic absorption of around 15% versus the monolayer MeO-2PACz more which in turn lowers the J_{SC} as shown in Supplementary Fig. 15b. The poly(3,4-ethylenedioxythiophene) polystyrene sulfonate (PEDOT:PSS) by itself did not work well with the co-evaporated $FAPbI_3$ recipe, which could potentially be due to the hydrophobicity[29] or surface property of PEDOT:PSS itself which could affect the adhesion or "sticking capability" of the FAI

during the evaporation process as well as the way the $FAPbI_3$ crystallises. It is likely that further optimisation would be required for the co-evaporated $FAPbI_3$ to work well with PEDOT:PSS. Another possibility could be due to energy band misalignment, although this often manifests as an S-shaped curve, which is not the case here as shown in Supplementary Fig. 15e. From the results, we thus adopted the bilayer combination of PEDOT:PSS/PTAA[30,31] as the third alternative. This bilayer PEDOT:PSS/PTAA indeed worked well, however it came at the expense of higher parasitic absorption of around 25% compared to MeO-2PACz as seen in Supplementary Fig. 15b where the average J_{SC} for the MeO-2PACz vs PEDOT:PSS/PTAA was around 20 vs 15 mA/cm^2 respectively.

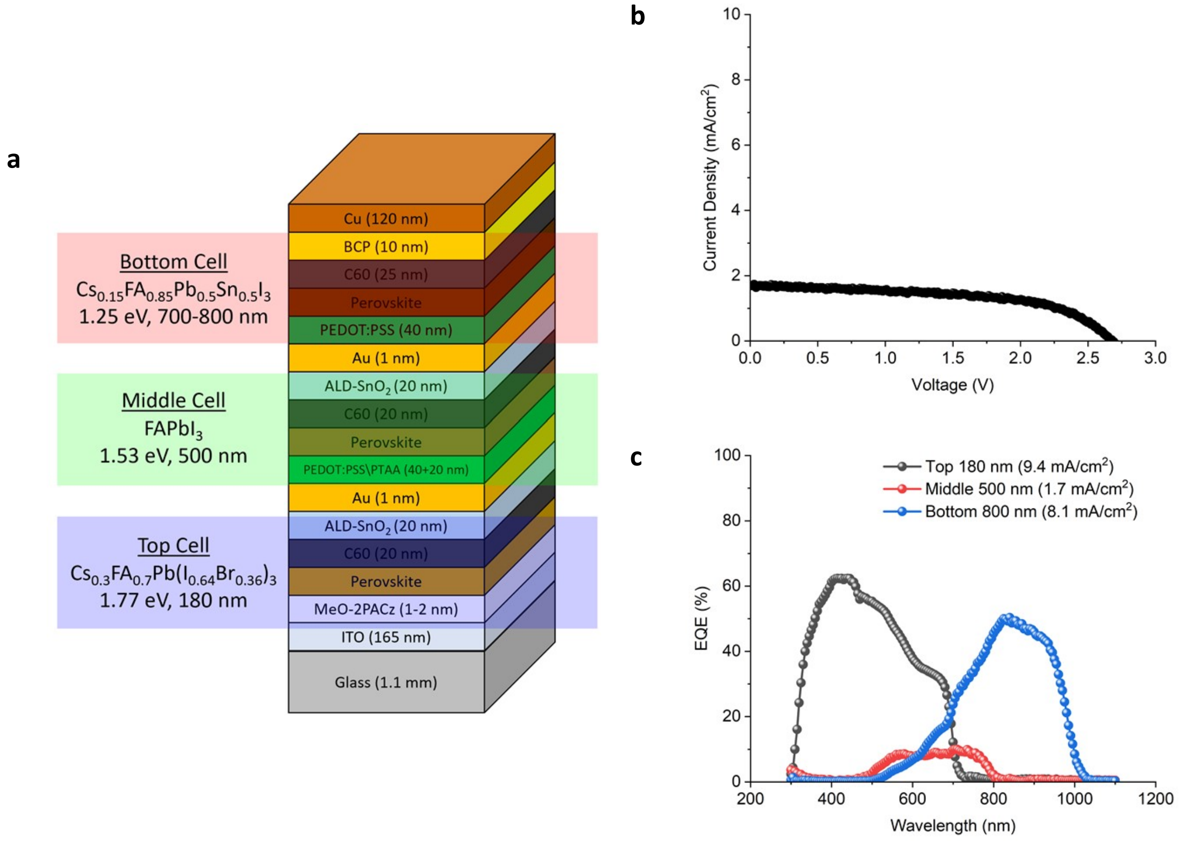

Supplementary Fig. 16 First monolithic PPP triple-junction solar cell batch results. a. Schematic illustration of the device stack, **b.** *J-V* (V_{OC} = 2.68 V, J_{SC} = 1.69 mA/cm^2, FF = 55.9%, PCE = 2.54%, and **c.** EQE of the subcells including the integrated EQE-J_{SC} values.

The first batch of triple-junction PPP devices was clearly middle subcell limited with a J_{SC} of only 1.7 mA/cm^2 as shown in Supplementary Fig. 16b,c both in *J-V* and EQE. This was mainly due to the thin unoptimized middle subcell thickness, but also reduced slightly due to the heavily parasitically absorbing PEDOT:PSS/PTAA layer.

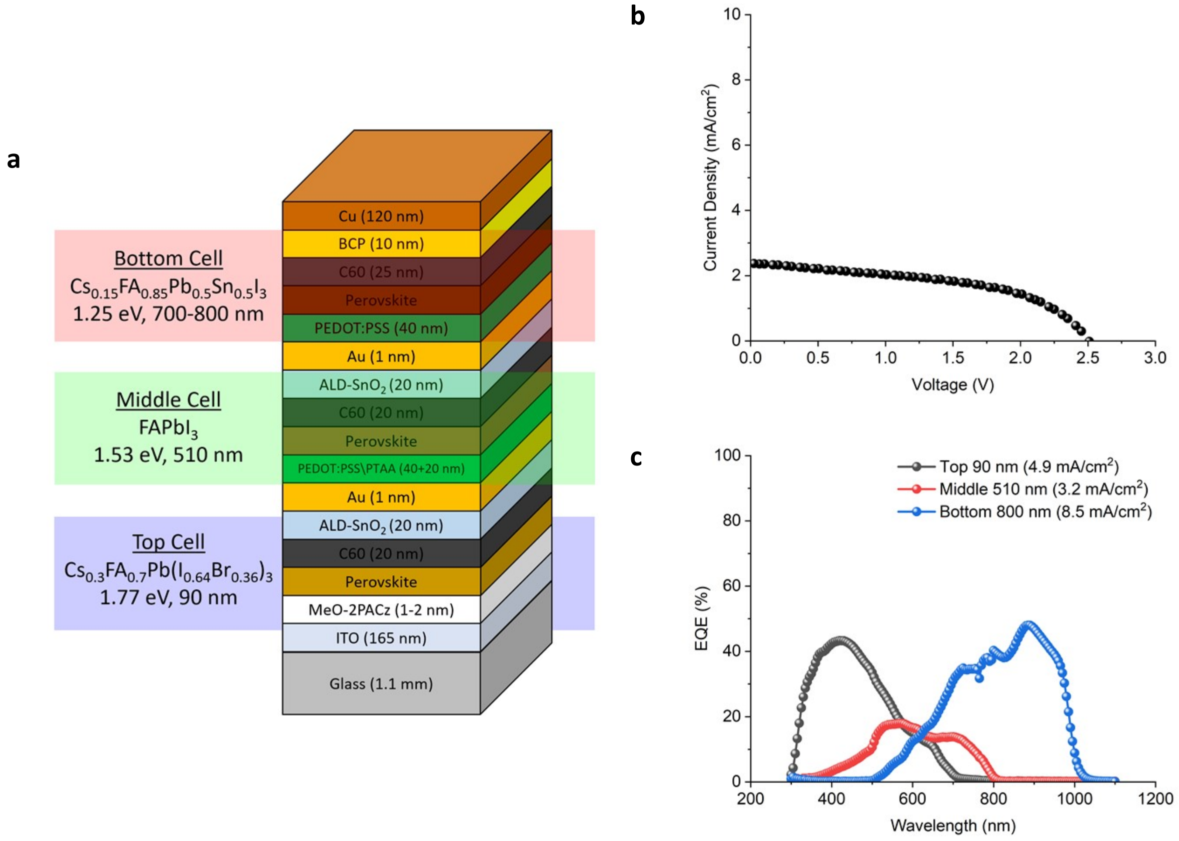

Supplementary Fig. 17 | Second monolithic PPP triple-junction solar cell batch results. a. Schematic illustration of the device stack, **b.** *J-V* (V_{OC} = 2.51 V, J_{SC} = 2.39 mA/cm^2, FF = 49.6%, PCE = 2.97%, and **c.** EQE of the subcells including the integrated EQE-J_{SC} values.

This batch had a significantly reduced top cell thickness from 180 nm to 90 nm, which improved the J_{SC} from the middle device. Representative devices showed that the EQE-J_{SC} was boosted from 1.7 to 3.2 mA/cm^2. This is still far from an ideal current-matched PPP triple-junction solar cell, but does demonstrate the importance of perovskite absorber thickness management in the design of triple-junction devices.

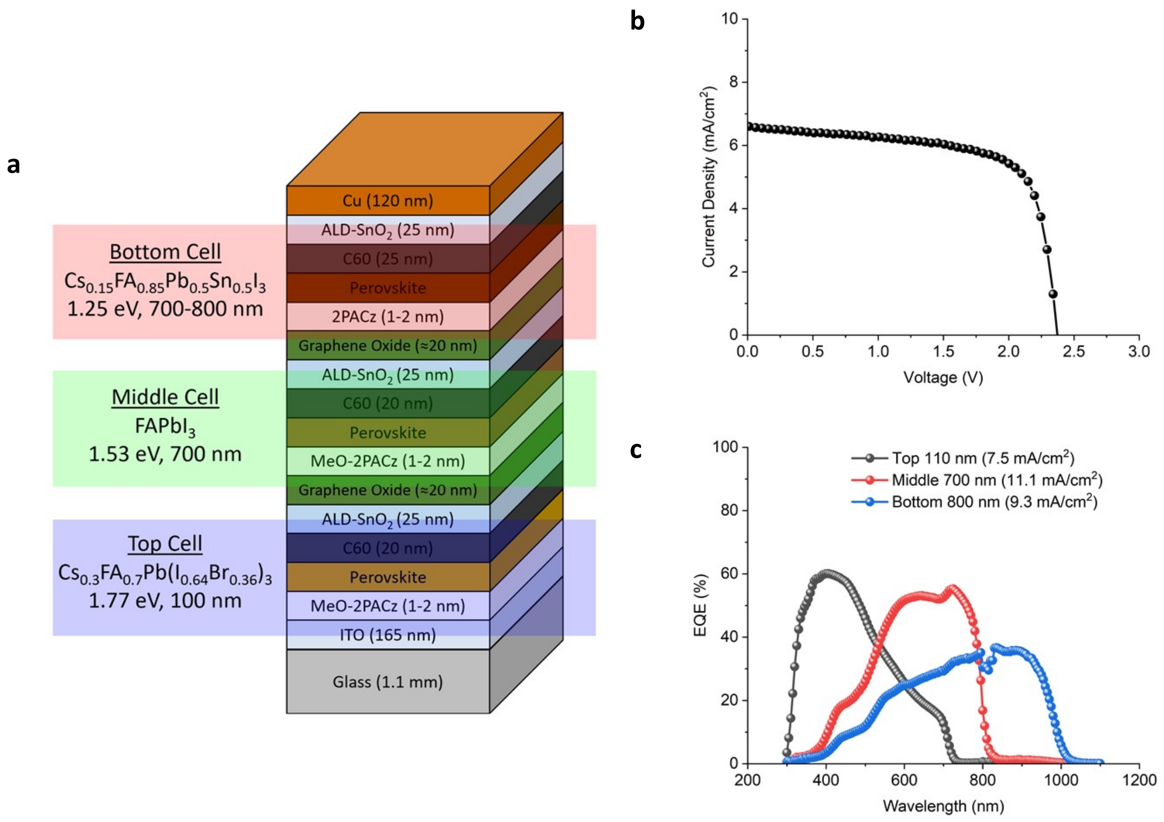

Supplementary Fig. 18| Third monolithic PPP triple-junction solar cell batch results (fabricated in May 2023). a. Schematic illustration of the device stack, **b.** *J-V* (V_{OC} = 2.37 V, J_{SC} = 6.61 mA/cm^2, FF = 69.38%, PCE = 10.88%, and **c.** EQE of the subcells including the integrated EQE-J_{SC} values.

Supplementary Fig. 18 shows results for the third monolithic PPP triple-junction device batch. Here we exchanged the interconnection for the top-to-middle subcells from the thin 1 nm of Au to spincoated graphene oxide (GO) nanoparticles. Thus the overall interconnect structure evolved from ALD-SnO_x/Au/PEDOT:PSS/PTAA to ALD-SnO_x/GO/MeO-2PACz (top-middle) subcell and ALD-SnO_x/Au/PEDOT:PSS to ALD-SnO_x/GO/2PACz (middle-bottom) subcell, to reduce the parasitic absorption of light passing through the device stack. What is more important to point out is that we were able to optimise the absorber thickness by thermally co-evaporating the top middle subcells so that they had thicknesses of 110 and 700 nm respectively as shown in Supplementary Fig. 18a. This result, although not perfect, demonstrated a significant improvement compared to the suboptimal devices shown in the two earlier batches. In the ideal case, the aim is to achieve a current-matched

maximum of approximately 9.5-10 mA/cm^2, given that our best single-junction Pb-Sn perovskite solar cells could achieve a J_{SC} of around 30.5 mA/cm^2 as shown in Supplementary Fig. 12a.

Supplementary Table 3 | Summary of the monolithic PPP triple-junction solar cell photovoltaic characteristics fabricated from Aug 22 to Jul 23

Date	Best Device *J-V* Parameters				EQE-J_{SC} (mA/cm^2)		
	V_{OC}	J_{SC} (mA/cm^2)	FF (%)	PCE (%)	Top	Middle	Bottom
Aug-22	2.68	1.69	55.88	2.54	9.4	1.7	8.1
Apr-23	2.51	2.39	49.60	2.97	4.9	3.2	8.5
May-23	2.37	6.61	69.38	10.88	7.5	11.1	9.3
Jul-23	2.38	9.27	71.56	15.77	9.6	9.3	9.0

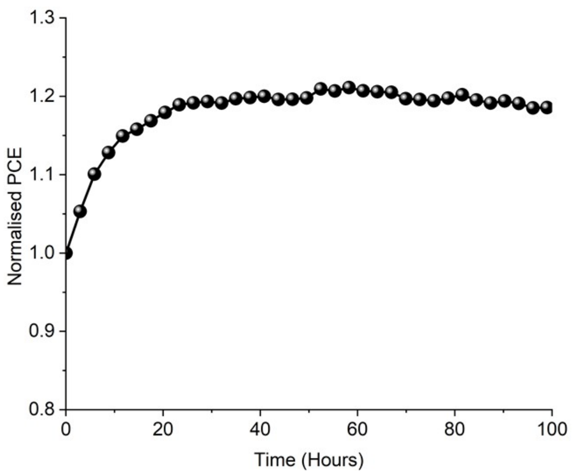

Supplementary Fig. 19 | Stability test. MPP tracking of the all-perovskite triple-junction solar cell with encapsulation for 100 h in air under full simulated AM1.5 solar illumination (100 mW cm^{-2}) held at 25 °C without ultraviolet filter.

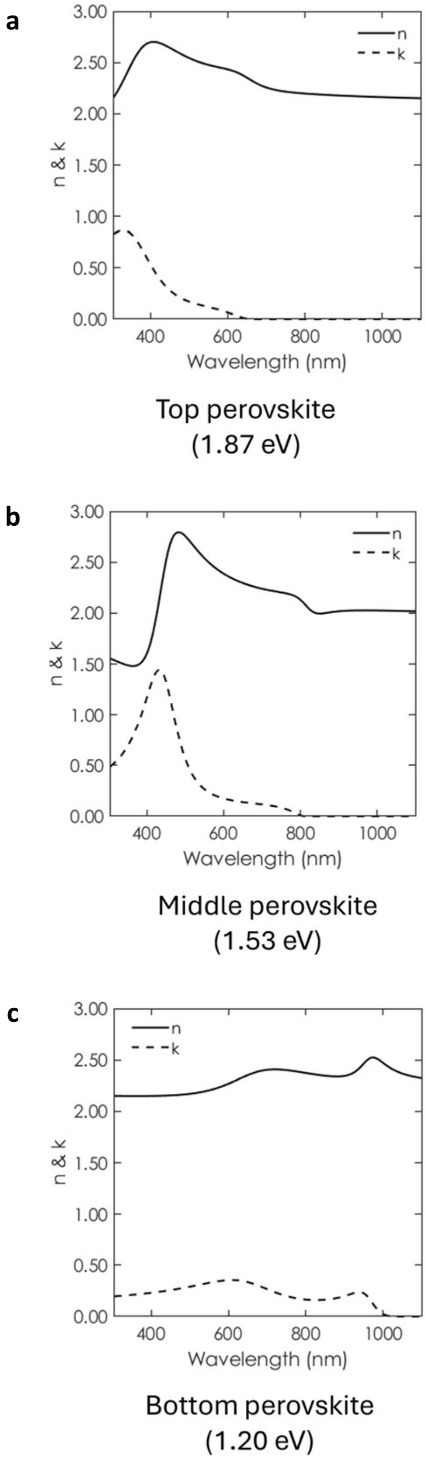

Supplementary Fig. 20 | Simulated complex refractive index data. Real, *n* (solid line) and imaginary, *k* (dashed line) parts of the complex refractive index of the **a.** top subcell **b.** middle subcell, and **c.** $Cs_{0.25}FA_{0.75}Pb_{0.5}Sn_{0.5}I_3$ (bottom) perovskites used in modelling of the simulated fully optimised perovskite triple-junction solar cell in Fig. 3a-c of the main text.

References

1. PV Education | Open-Circuit Voltage. https://www.pveducation.org/pvcdrom/solar-cell-operation/open-circuit-voltage.
2. Hörantner, M. T. *et al.* The Potential of Multijunction Perovskite Solar Cells. *ACS Energy Lett.* **2**, 2506–2513 (2017).
3. Bowman, A. R. *et al.* Relaxed current matching requirements in highly luminescent perovskite tandem solar cells and their fundamental efficiency limits. *ACS Energy Letters* **6**, 612–620 (2021).
4. Green, M. A. & Ho-Baillie, A. W. Y. Pushing to the Limit: Radiative Efficiencies of Recent Mainstream and Emerging Solar Cells. *ACS Energy Lett.* **4**, 1639–1644 (2019).
5. Brenes, R., Laitz, M., Jean, J., deQuilettes, D. W. & Bulović, V. Benefit from Photon Recycling at the Maximum-Power Point of State-of-the-Art Perovskite Solar Cells. *Phys. Rev. Applied* **12**, 014017 (2019).
6. deQuilettes, D. W. *et al.* Maximizing the external radiative efficiency of hybrid perovskite solar cells. *Pure and Applied Chemistry* **92**, 697–706 (2020).
7. Hoke, E. T. *et al.* Reversible photo-induced trap formation in mixed-halide hybrid perovskites for photovoltaics. *Chem. Sci.* **6**, 613–617 (2015).
8. Slotcavage, D. J., Karunadasa, H. I. & McGehee, M. D. Light-Induced Phase Segregation in Halide-Perovskite Absorbers. *ACS Energy Lett.* **1**, 1199–1205 (2016).
9. deQuilettes, D. W. *et al.* Photo-induced halide redistribution in organic–inorganic perovskite films. *Nat Commun* **7**, 11683 (2016).
10. Bischak, C. G. *et al.* Origin of Reversible Photoinduced Phase Separation in Hybrid Perovskites. *Nano Lett.* **17**, 1028–1033 (2017).
11. Draguta, S. *et al.* Rationalizing the light-induced phase separation of mixed halide organic–inorganic perovskites. *Nat Commun* **8**, 200 (2017).
12. Unger, E. L. *et al.* Roadmap and roadblocks for the band gap tunability of metal halide perovskites. *J. Mater. Chem. A* **5**, 11401–11409 (2017).

13. Yang, T. C.-J., Fiala, P., Jeangros, Q. & Ballif, C. High-Bandgap Perovskite Materials for Multijunction Solar Cells. *Joule* **2**, 1421–1436 (2018).
14. Belisle, R. A. *et al.* Impact of Surfaces on Photoinduced Halide Segregation in Mixed-Halide Perovskites. *ACS Energy Lett.* **3**, 2694–2700 (2018).
15. Brennan, M. C., Draguta, S., Kamat, P. V. & Kuno, M. Light-Induced Anion Phase Segregation in Mixed Halide Perovskites. *ACS Energy Lett.* **3**, 204–213 (2018).
16. Mahesh, S. *et al.* Revealing the origin of voltage loss in mixed-halide perovskite solar cells. *Energy Environ. Sci.* **13**, 258–267 (2020).
17. Chen, Z., Brocks, G., Tao, S. & Bobbert, P. A. Unified theory for light-induced halide segregation in mixed halide perovskites. *Nat Commun* **12**, 2687 (2021).
18. Motti, S. G. *et al.* Phase segregation in mixed-halide perovskites affects charge-carrier dynamics while preserving mobility. *Nat Commun* **12**, 6955 (2021).
19. Wang, Z. *et al.* Suppressed phase segregation for triple-junction perovskite solar cells. *Nature* **618**, 74–79 (2023).
20. McMeekin, D. P. *et al.* Solution-Processed All-Perovskite Multi-junction Solar Cells. *Joule* **3**, 387–401 (2019).
21. Boccard, M. & Ballif, C. Influence of the Subcell Properties on the Fill Factor of Two-Terminal Perovskite–Silicon Tandem Solar Cells. *ACS Energy Lett.* **5**, 1077–1082 (2020).
22. Gaillet, M. New Amorphous Dispersion Formula.
23. Ávila, J. *et al.* High voltage vacuum-deposited $CH_3NH_3PbI_3$–$CH_3NH_3PbI_3$ tandem solar cells. *Energy Environ. Sci.* **11**, 3292–3297 (2018).
24. Xiao, K. *et al.* All-perovskite tandem solar cells with 24.2% certified efficiency and area over 1 cm2 using surface-anchoring zwitterionic antioxidant. *Nat Energy* **5**, 870–880 (2020).
25. König, T. A. F. *et al.* Electrically Tunable Plasmonic Behavior of Nanocube–Polymer Nanomaterials Induced by a Redox-Active Electrochromic Polymer. *ACS Nano* **8**, 6182–6192 (2014).

26. McPeak, K. M. *et al.* Plasmonic Films Can Easily Be Better: Rules and Recipes. *ACS Photonics* **2**, 326–333 (2015).

27. Komisar, D. A. *et al.* Optical properties of thin graphene oxide films and their biosensing applications. *J. Phys.: Conf. Ser.* **1461**, 012068 (2020).

28. Al-Ashouri, A. *et al.* Conformal monolayer contacts with lossless interfaces for perovskite single junction and monolithic tandem solar cells. *Energy & Environmental Science* **12**, 3356–3369 (2019).

29. Lu, B. *et al.* Pure PEDOT:PSS hydrogels. *Nat Commun* **10**, 1043 (2019).

30. Wang, J. *et al.* 16.8% Monolithic all-perovskite triple-junction solar cells via a universal two-step solution process. *Nat Commun* **11**, 5254 (2020).

31. Xiao, K. *et al.* Solution-processed monolithic all-perovskite triple-junction solar cells with efficiency exceeding 20%. *ACS Energy Letters* **5**, 2819–2826 (2020).
